# Accessing Multiple Phases via Thermodynamic or Kinetic Pathways: The Impact of Bivalent Ferrocene Spacers on 2D Hybrid Perovskite Formation

**DOI:** 10.1021/acsami.5c14485

**Published:** 2025-10-24

**Authors:** Melina Dahlke, Yaşar Krysiak, Marvin Treger, Carolin König, Sebastian Polarz

**Affiliations:** † Institute of Inorganic Chemistry, 26555 Leibniz University of Hannover, Callinstraße 9, 30167 Hannover, Germany; ‡ Cluster of Excellence PhoenixD, 26555 Leibniz University of Hannover, Welfengarten 1A, 30167 Hannover, Germany; § Insitute of Physical and Electrochemistry, 26555 Leibniz University of Hannover, Callinstraße 3A, 30167 Hannover, Germany

**Keywords:** 3D electron diffraction, hybrid material, Dion−Jacobson, Ruddlesden−Popper, band-gap

## Abstract

Many semiconductor technologies require interfacing materials with different properties. 2D hybrid perovskites are one of the most promising candidates, combining the advantages of organic and inorganic layers. The networks of linked metal-halide octahedra with voids filled by organic counterions have proven high variability and can be tailored to specific applications. The geometric and electronic setup of the organic linker molecule between inorganic layers affects the crystal structure and the overall optoelectronic properties. Monoamines typically form bilayers in so-called Ruddlesden–Popper phases (RPs), while bisamines allow for making Dion–Jacobson phases (DJs), with only a monolayer directly bridging the inorganic layers. Therefore, it would be highly interesting if one could compare RPs to DJs directly to each other, meaning that they have been prepared using exactly the same organic linker molecule, which is the aim of the study presented here. Because of the potential interaction of π-conjugated compounds with the electronic system of the semiconductor, we have selected a special linker here: a divalent ferrocene derivative containing one primary amine attached to each of the cyclopentadienyl rings. These linkers form novel quasi-DJs, and their structure was determined by 3D electron diffraction and density functional theory. We found that by different crystallization kinetics, two quasi-DJ variants and even RPs can be obtained from the same spacer molecule. It takes time for the ferrocene-based linker to adjust to a particular conformation, giving the system also time to form octahedral connections other than the classic DJ/RP corner-sharing. The different octahedral linkages, ranging from face- to corner-sharing, have a significant influence on the optoelectronic properties. Thus, with a change of the synthesis conditions, it is possible to change the structure and the properties of the same educts. Our study also enables the first direct comparison of quasi-DJ and RP phases by achieving both with the same spacer molecule.

## Graphical abstract

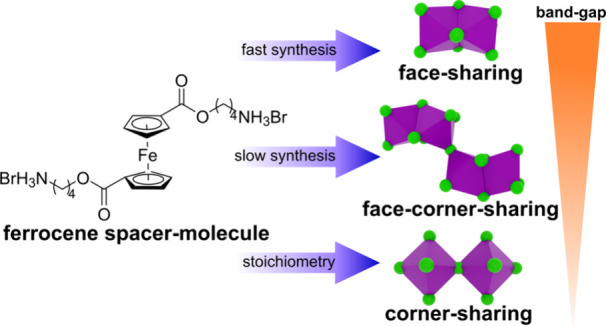

## 1 Introduction

In the last decade, the rapid advancement of semiconductor technologies has fueled an increasing demand for novel, more efficient materials.^1,2^ Because producing many established semiconductors with silicon as the prime example consumes significant resources, most importantly energy,^2–5^ finding a substitute would represent a breakthrough if it could be processed from a liquid phase at room temperature and ambient pressure. Therefore, enormous attention has been invested in research on the so-called hybrid perovskites.^6,7^ Hybrid perovskites are organic–inorganic semiconductors and structural derivatives of purely inorganic perovskites, in which at least one ion is exchanged by an organic ion.

The inorganic perovskites usually have a cubic or related structure with the sum formula of ABX_3_. The [BX_6/2_]^*Y*−^ octahedra form a three-dimensional network via shared corners with an A-cation in between. The A-cation site plays a crucial role in the formation of the perovskite structure, and a change in size may not only lead to a distortion of the octahedra to increase the space for the cation^8^ but also to a change in the connection patterns of the octahedra.^9,10^ This interplay of size and structure was highlighted by Sto̷len and co-workers^11^ in 2007, who demonstrated the change of connectivity in manganate perovskite minerals with the cations Ca, Sr, and Ba. With the increasing size of the A-cation, the connection of the octahedra changed from corner- to face-sharing. The structural diversity of perovskite-like materials manifests itself in unique optical properties. Thus, perovskite materials display adjustable properties for various specific applications.

The discovery of hybrid perovskites marked another major leap toward structural diversity. The A-cation is now substituted by a small organic molecule such as methylammonium.^12^ These organic–inorganic hybrid systems opened the door to novel functionalities and properties. In the beginning, major attention was on solar cell research because of the high power conversion efficiencies of the hybrid perovskites with the ordinary 3D crystal structure.^13–15^ The field evolved rapidly; interest became broader, and in particular, the possibilities in using more complex organic cations were intriguing. Above a critical size, two-dimensional (2D) layered hybrid perovskites form,^16^ which proved to have numerous advantageous properties compared to the 3D systems.^17,18^ 2D hybrid perovskites typically form Ruddlesden–Popper phases (RPs). RPs contain a bilayer of monovalent spacer cations (A_2_BX_3_), which are connected via the so-called van der Waals (vdW) gap.^19,20^ The gap hampers electronic delocalization perpendicular to the layers, and thus, charge transport is reduced in this direction. A vdW gap can be avoided in the so-called Dion–Jacobson phases (DJs). DJs (ABX_3_) are prepared using a bridging divalent, typically diammonium, spacer cation. The organic monolayer leads to enhanced stability and a better electronic communication between the inorganic layers compared to RPs,^21,22^ whereas a direct comparison is difficult when two chemically distinct linker molecules have to be used. Electronic communication in this case means the charge transport and, respectively, the charge carrier dynamics, between inorganic layers, through the organic layer. Simulations show an increased delocalization of DJ phases compared to that of RPs. This communication might be further improved by the introduction of aromatic or electrostatically more active spacer molecules.^23^ To implement such spacer molecules into DJs is a challenge in itself. Entropic effects reduce the thermodynamic stability as the conformational flexibility of the linker molecules is reduced significantly because of the molecular rigidity of those systems, which pose even more constraints to the molecular conformation and arrangement. In addition, if one wants to compare RPs directly with DJs, then one has to make both phases essentially with the same organic spacer. The latter has never been achieved and represents the goal of the current studies.

In a previous work, we reported a new type of RP like hybrid perovskites using a monoamine-modified ferrocene (Fc) entity,^24^ which we called ferrovskites. Ferrocene is an exceptional molecular switch, which can be modified in various ways.^25,26^ Its stability and reliable redox switching have great potential for the synthesis of semiconductors with changing properties. The synthesized ferrovskites were structurally investigated, and X-ray photoelectron spectroscopy (XPS) showed that up to 50% of the Fc in the bilayers could be oxidized to Fc^+^ (ferrocenium), with notable consequences for the optoelectronic properties of the materials. The results indicated that there is indeed electronic communication in the form of charge transfer between the lead halide layers and the Fc layers. Obviously, a monoaminated Fc is not capable of forming DJs, which is why we became interested in bis-aminated Fc spacers. With a rigid aromatic system and flexible side chains, ferrocene possess all properties needed to arrange itself with a structure-directing effect in DJ phases.

The paper is organized as follows: Divalent ferrocene-based spacer cations were synthesized and used to crystallize hybrid perovskites to evaluate the DJ phase formation. We varied the synthesis conditions to assess the structure-directing influence of a single ferrocene spacer molecule in DJ phases. The resulting phases were structurally characterized by 3D electron diffraction and density functional theory (DFT) calculations, and correlations between structural features and material properties were established.

## 2 Experimental Section

### 2.1 Chemicals

Lead­(II) bromide (PbBr_2_, Sigma-Aldrich, 99.9% purity), 1,1′-ferrocene dicarboxylic acid (C_12_H_10_FeO_4_, alfa aesar, 97% purity), *N*-Boc-ethanolamine (C_7_H_15_NO_3_, Sigma-Aldrich, 98% purity), 3-(Boc-amino)-1-propanol (C_8_H_17_NO_3_, TCI Chemicals, 96% purity), 4-(Boc-amino)-1-butanol (C_9_H_19_NO_3_, TCI Chemicals, 97% purity), 5-(Boc-amino)-1-pentanol (C_10_H_21_NO_3_, TCI Chemicals, 97% purity), 6-(Boc-amino)-1-hexanol (C_11_H_23_NO_3_, Sigma-Aldrich, 98% purity), 4-(dimethylamino) pyridine (DMAP, C_7_H_10_N_2_, Sigma-Aldrich, 98% purity), *N*-(3-(dimethylamino)­propyl)-*N*′-ethylcarbodiimide hydrochloride (EDC-HCl, C_8_H_17_N_3_–HCl, TCI Chemicals, 98% purity), trifluoroacetic acid (TFA, C_2_HO_2_F_3_, Sigma-Aldrich, 99% purity), hydrobromic acid (HBr, 48 wt % in H2O, Sigma-Aldrich), dichloromethane (DCM, ≥99.5% purity, Carl Roth), toluene (≥99.5% purity, Carl Roth), ethanol (EtOH, ≥98% purity, Carl Roth), acetone (≥99.5% purity, Carl Roth), 1,4-dioxane (≥99.5% purity, Carl Roth), ammonium chloride (NH_4_Cl, ≥99.5% purity, PanReac AppliChem), sodium hydrogen carbonate (NaHCO_3_, ≥99.5% purity, Carl Roth), sodium chloride (NaCl, ≥99.5% purity, Carl Roth), and magnesium sulfate (MgSO_4_, ≥98% purity, Carl Roth) were used.

### 2.2 Synthesis of the Divalent Ferrocene Dicarboxylate Dibutyl Amine Fc­(C_*n*_N)_2_

The scheme of the synthesis can be found in Figure S1. For the esterification, 301.5 mg of 1,1′-ferrocene dicarboxylic acid (1.1 mmol, 1 equiv) was dissolved in 50 mL of dichloromethane (DCM) using ultrasonication. Then, the *N*-Boc-ethanol-amine (2.2 mmol, 2 equiv) [respective Boc-protected amines: 3-(Boc-amino)-1-propanol, 4-(Boc-amino)-1-butanol, 5-(Boc-amino)-1-pentanol, 6-(Boc-amino)-1-hexanol], 317.6 mg of 4-(dimethylamino) pyridine (DMAP) (2.6 mmol, 2.36 equiv), and 498.4 mg of *N*-(3-dimethyl-aminopropyl)-*N*′-ethylcarbodiimide hydrochloride (EDC–HCl) (2.6 mmol, 2.36 equiv) were added. After a few minutes, the reactants dissolved, and the solution turned red. The solution was then heated to 50 °C under reflux for 18 h. The red solution was washed three times with a saturated NH_4_Cl solution, two times with a saturated NaHCO_3_ solution, and two times with brine to remove excess catalysts. The mixture was dried over MgSO_4_ and filtered, and the solvent was removed under vacuum to give an orange oil.

To remove the Boc-protecting group, the oil was redissolved in 50 mL of DCM, and 5 mL of trifluoroacetic acid (66 mmol, 60 equiv) was added. The solution was stirred for 1 h at 50 °C under reflux. The solvent was removed, and the orange oil was co-evaporated with 40 mL of toluene twice.

For the protonation of the ammonium group, the oil was dissolved in 40 mL of 1,4-dioxane, and 0.24 mL of HBr (48% in H_2_O, 4.4 mmol, 4 equiv) was added to gain the ammonium bromide salt. After 2 min of stirring, a solid started to precipitate. The mixture was stirred for another hour before the solvent was removed, giving an orange solid.

The product was purified by recrystallization. The orange solid was dissolved in 5 mL of ethanol and reprecipitated in diethyl ether (Et_2_O). The mixture was kept in a refrigerator overnight. The solvent was decanted, and the orange powder was dried under vacuum. The product was characterized by ^1^H NMR and ^13^C NMR (Figures S2–S6).

### 2.3 Antisolvent Synthesis of the Perovskite Phase (Fast Synthesis)

The precursor solution for the antisolvent synthesis of the different perovskite phases was synthesized by dissolving PbBr_2_ (0.05 mmol, 1 equiv) and the spacer cation Fc­(C_*n*_N)_2_ (0.05 mmol, 1 equiv) in 0.5 mL of dried DMF. The mixture was treated in an ultrasonic bath for 5 min and was then quickly added to 30 mL of DCM under vigorous stirring. The suspension was stirred for 1 h before the orange solid was centrifuged and washed with DCM once and with EtOH twice. The solid was dried under a vacuum. The resulting orange powder was stored under a nitrogen atmosphere.

### 2.4 Antisolvent Vapor-Assisted Perovskite Crystallization (Slow Synthesis)

The precursor solution was prepared by dissolving PbBr_2_ (0.05 mmol, 1 equiv) and the spacer cation (0.05 mmol, 1 equiv) in 0.5 mL of dried DMF for the quasi-DJ phase and 2 equiv of spacer cation for the RP phase. The mixture was treated in an ultrasonic bath for 5 min. The precursor solution, stored in a 3 mL vial, was placed in a 50 mL vial with 10 mL of DCM in it. The 50 mL vial was left closed for 2 days at room temperature until the precursor vial (3 mL) was filled up by diffusion. The perovskite was crystallized by slow diffusion of the vapor of the antisolvent into the precursor solution. The solid was washed with DCM and dried under reduced pressure, and the orange powder was stored under a nitrogen atmosphere.

This method was originally used by Bakr and co-workers^27^ as a single crystal synthesis and allowed the formation of a perovskite within 2 to 4 days, depending on the temperature/diffusion rate.

### 2.5 3D Electron Diffraction

Three-dimensional electron diffraction (3D ED) measurements of kinetically and thermodynamically synthesized C_4_ ferrovskite phases were carried out using a Hitachi HT7800 transmission electron microscope (TEM) of a Hitachi HT7800 operating at 120 kV. The powder samples were dispersed in toluene using an ultrasonic bath. The particles took 1–5 min to disperse completely without the occurrence of any heat generation and were then drop-cast on a holey carbon-coated copper grid. TEM images and ED patterns were recorded with an EMSIS Xarosa CMOS camera (14-bit, 5120 × 3840 pixels). 3D ED data were collected using the authors' own acquisition module, *eHermelin*, developed for SerielEM.^28^ A condenser aperture of 50 μm and mild illumination settings (F-zoom, spot number 1) were used. The crystals were continuously tilted in a maximum range of 100° with an angular speed of 1.5454°. The PETS2.0 software package^29^ was used for 3D ED data processing. Structure solution and refinement were performed with the software JANA2020.^30^ Bond distances and angles of the spacer cation Fc­(C_4_Br)_2_ were restrained to the structural motifs found in the CSD database.

### 2.6 Powder X-ray Diffraction and Rietveld Refinement

Measurements of the powder samples were performed using an X-ray diffractometer with a Debye–Scherrer setup (StadiP by Stoe). Data were collected in eight ranges with a step size of 0.015° over a 2θ range of 2°–100°, using monochromatic CuKα_1_ radiation (λ = 1.54059 Å). The diffractometer was equipped with a Mythen 1K detector (Dectris) with an angular range of 12.5°.

The Rietveld refinement was carried out using the TOPAS Version 6 software.^31^ Bond distances and angles of the spacer cation Fc­(C_4_Br)_2_ were restrained to the structural motifs found in the CSD database.

### 2.7 DFT Calculations

Kohn–Sham DFT calculations were performed using the CASTEP code (version 25.11), employing a plane-wave basis set in combination with pseudopotentials.^32–34^ The convergence of the plane-wave kinetic energy cutoff and the Monkhorst–Pack (MP) grid for Brillouin zone sampling was examined with respect to the band gap (Figures S13 and S14).^35^ The PBEsol exchange–correlation (XC) functional was used to fully relax the structures using “on-the-fly” generated ultrasoft pseudopotentials with an SCF convergence criterion of 5.0 × 10^–7^ eV per atom.^36^ For the kinetic ferrovskite phase, the Tkatchenko–Scheffler dispersion correction was used in addition.^37,38^ Applying a low-memory Broyden–Fletcher–Goldfarb–Shanno (LBGFS) algorithm, structures with an energy change of less than 5.0 × 10^–6^ eV per atom and a maximal force of 0.04 eV Å^–1^ were obtained.^39–41^ The convergence threshold of the stress and maximal atom displacement was 0.02 GPa and 5.0 × 10^–4^ Å, respectively. Using the optimized models, single-point PBEsol DFT calculations were performed to obtain the band structures and the corresponding density of states (DOS). The DOS and projected DOS (PDOS) were calculated using OptaDOS.^42^

### 2.8 UV–Vis Spectroscopy Measurements

The UV–vis absorption spectra were recorded in toluene (absorption >4.43 eV), which shows absorption bands in a different region than the band gaps of the perovskites. As it is a nonpolar solvent, the perovskites are stable and easy to disperse. 2 ml of a low-concentration dispersion was measured in a quartz cuvette. UV–vis measurements of particles in dispersion and the spacer cations in solution were obtained with an Agilent 8453 Cary 4000.

### 2.9 Other Characterizations

SEM images and energy-dispersive X-ray (EDX) spectroscopy of particles on carbon tape were obtained by using a Hitachi Regulus SU8200. Photoelectron spectroscopy (on air; PESA) of drop-cast particles on a glass substrate was acquired with a Riken Keiki AC-2 photoelectron spectrometer. Liquid NMR measurements were obtained with a JEOL 400 MHz spectrometer.

## 3 Results and Discussion

### 3.1 Divalent Ferrocene Amines as the Novel Spacer

Symmetric, bis-functionalized 1,1′-[(2-aminoalkoxy)­carbonyl]­ferrocene derivatives ((Fc­(COO­(CH_2_)_*n*_NH_2_)_2_); *n* = 3–6) and their ammonium bromide salts, denoted as Fc­(C_*n*_Br)_2_, were synthesized by an esterification of the 1,1′-ferrocene dicarboxylic acid and the respective BOC-amine, followed by the deprotection and hydrobromination of the amines (Supporting Information Figure S1; see also the experimental part). Nuclear magnetic resonance (NMR) spectroscopy and further characterization data of the compounds are given in the Supporting Information (Figures S2–S5) and indicate the success in the synthesis of the new spacer molecules.

### 3.2 Two-Dimensional Hybrid Perovskites Prepared Using Fc­(C_4_Br)_2_ as a Linker

An antisolvent approach was used to prepare the ferrovskites. First, we concentrate on the materials obtained by using Fc­(C_4_Br)_2_ as a linker. The spacer cation and PbBr_2_ were employed in 1:1 stoichiometry, aiming for DJs that precipitate as a microcrystalline powder. Different reaction rates were realized by adding the antisolvent (dichloromethane; DCM) rapidly within seconds via a syringe or slowly within days via an evaporation-based method; see Supporting Information Figure S10. The resulting samples are named ″k” (k corresponds to kinetic) or ″e” (e corresponds to equilibrium) C_4_Ferov and were initially investigated by powder X-ray diffraction (PXRD; see Figure 1). One knows from research on 2D perovskites that the low-angle diffraction signal is indicative of the layer-to-layer distance *d* (interlayer spacing).^43^ The *d*-spacing can be calculated from the first reflection using Bragg’s equation. *d* depends on the size of the organic cations, and if there is a monolayer (DJ) or a bilayer (RP), as well as the arrangement of the inorganic octahedra. Given the extension of Fc­(C_4_Br)_2_ and the values we obtained in our previous work on RP ferrovskites^24^ using the monoamines FcC_*n*_Br, one can estimate that *d* ≈ 19 Å is indicative of an RP, and smaller values speak for DJ (Figure 1b). The small *d* values (12.26, 15.70 Å) for “k”- and “e”-C_4_Ferovs indicate that the required DJs could be obtained. Because the diffraction patterns are different (Figure 1), one must conclude that the crystal structures of the samples are variants.

**Figure 1:**
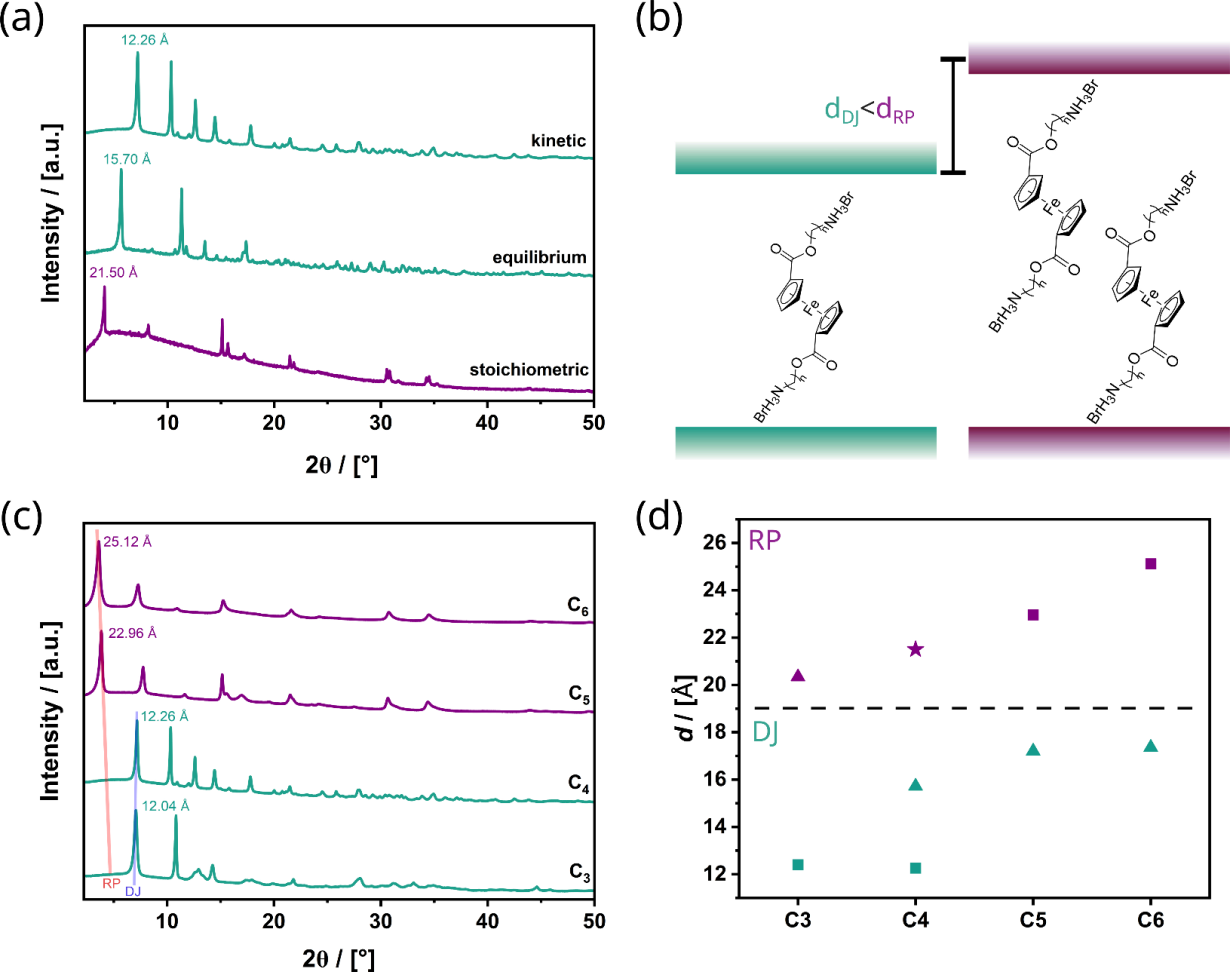
(a) PXRD patterns “k”- (top), “e”- (middle), and “s”-C_4_Ferov materials (bottom). (b) Schematic comparison of the higher interplanar distance *d* of Ruddlesden–Popper phases (purple) compared with the reduced space in Dion–Jacobson phases (turquoise). (c) PXRD patterns of “k”-C_*n*_Ferovs with *n* = 3–6. (d) Comparison of the interlayer spacing of the “k”-C_*n*_Ferovs (squares), “e”-C_*n*_Ferovs (triangles), and the third “s”-C_4_Ferovs phase (star) and their categorization into RP (purple) and DJ (turquoise) phases.

A more precise analysis and discussion of the crystal structures is needed. Both fast and slow crystallization methods delivered crystallites with sizes of only a few micrometers, which were not large enough for single-crystal analysis by X-ray diffraction (see Supporting Information Figure S9). As an alternative, we applied 3D electron diffraction (3D ED) as it can be performed on hybrid perovskite microcrystals, as shown in ref 24. Despite the pronounced sensitivity of the materials against the electron beam, we succeeded in determining the octahedral lead–bromide network and the iron positions of the ferrocene derivatives. Figure 2a,b shows exemplary, reciprocal space sections *(h*0*l)* for both DJ phases, which, on the one hand, illustrate that the particles diffract like single crystals and, on the other hand, demonstrate the difference of the lattices between the two monoclinic phases. Details regarding the 3D ED method can be found in the SI (Table S1).

**Figure 2:**
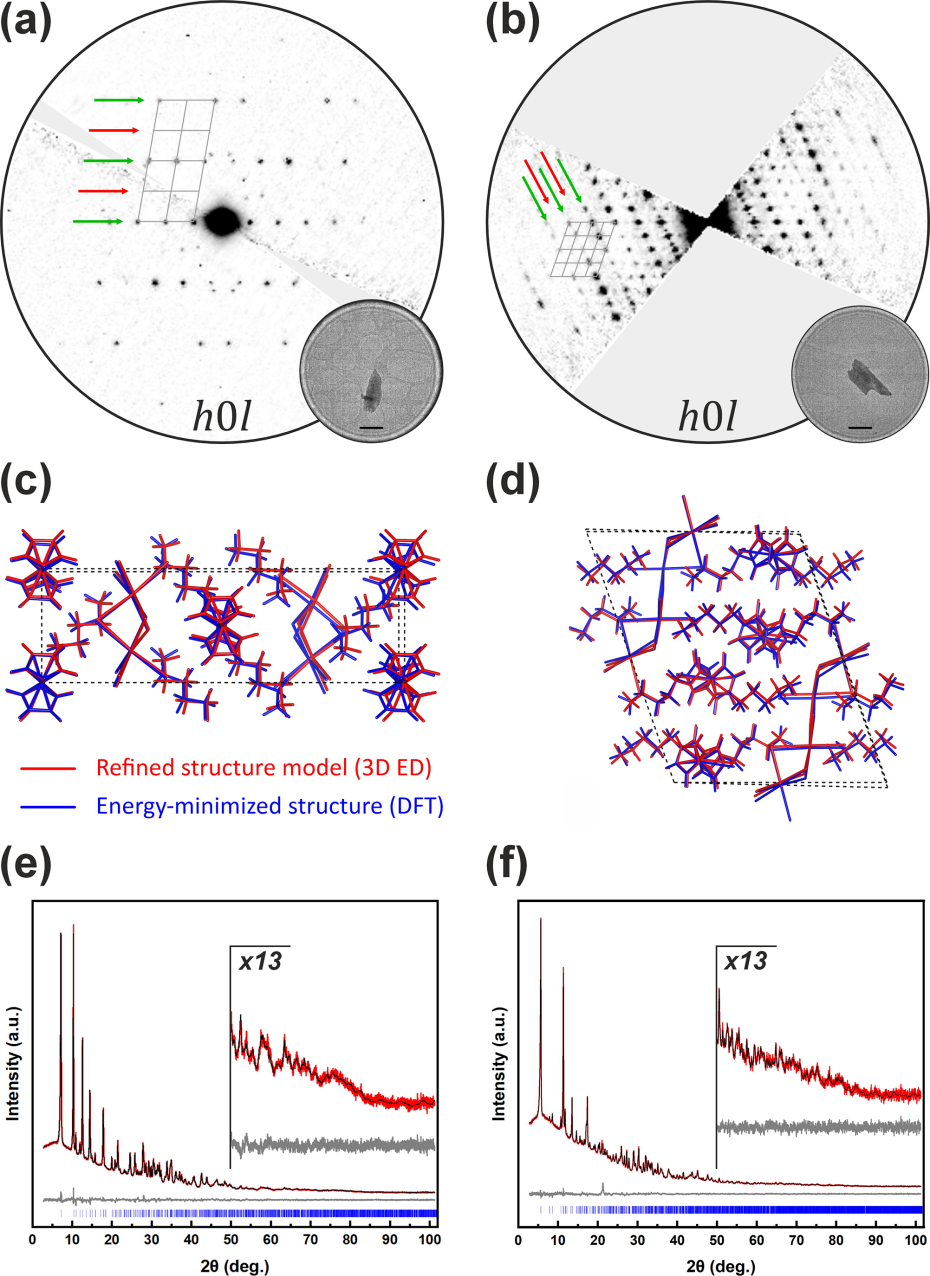
Exemplary reciprocal space sections *h*0*l* measured by 3D ED of (a) “k”-C_4_Ferov (*P*2_1_/*c*) and (b) “e”-C_4_Ferov (*P*2_1_/*n*). Forbidden and allowed zonal reflection conditions are marked with red or green arrow. The respective measured crystals are shown in the circular insets (scale bar: 1 μm). (c, d) Superposition of the experimental crystal structures of the (c) “k”-C_4_Ferov and (d) “e”-C_4_Ferov (in red) on the optimized structures (in blue) calculated by DFT. (e, f) Rietveld refinement plots (λ = 1.54059 Å) of the DJ ferrovskites (e) “k”- and (f) “e”-C_4_Ferov. Red line for measured intensities (*I*_O_), black line for the fitted profile (*I*_C_), and gray line for the difference (*I*_O_ – *I*_C_).

Despite the low total accumulated electron dose during acquisition (8–10 e/Å^2^), both phases decomposed rapidly in the electron beam. Furthermore, the crystallinity of each phase is relatively low. Consequently, the scattering power of the crystals of both phases was comparatively low, with reflections observed only up to 0.8 Å^–1^ (see sections in Figure S11). Because of that, crystal structure models of “k”-C_4_Ferov and “e”-C_4_Ferov were established through a four-step process. (1) In addition to the lead–bromide octahedra, the ferrocene units could be identified from the 3D ED experiment, and the cyclopentadienyl ligands (Cp) had to be completed. The side chain of the Cp was then added by hand. (2) The completed crystal structure models were fully optimized using DFT methods (Figure 2c,d, Table S2, Figures S12–S14). (3) Structure refinements of the geometry-optimized phases against 3D ED data converged for both the “k”-C_4_Ferov and “e”-C_4_Ferov using restraints for the ferrocene ligands (for more details, see Table S1). The root-mean-square deviation (RMSD) of the coordinates of all atoms (except H atoms) is less than 0.04 Å, which means that the completed crystal structures of both phases are very likely correct. (4) The overall high quality of the Rietveld refinements (Figure 2e,f) confirms that the crystal structures of both phases describe the entire powder sample. All methods combined lead to a reliable structure determination of the “e”- and “k”-C_4_Ferov materials. The crystallographic details are summarized in Table 1.

**Table 1:** Crystallographic Data and Structural Details of the “k”- and “e”-C_4_Ferov DJ Phases Determined by a Combination of 3D ED Measurements, DFT Calculations and Rietveld Refinement.

C_4_ ligand:	“k”-C_4_Ferov	“e”-C_4_Ferov
empirical formula	[Fc(C_4_)_2_]Pb_2_Br_6_	[Fc(C_4_)_2_]_2_Pb_3_Br_10_
crystal system	monoclinic	monoclinic
space group	*P*2_1_/*c*	*P*2_1_/*n*
*a* [Å]	8.6374(3)	21.7909(1)
*b* [Å]	24.3504(1)	8.4317(3)
*c* [Å]	7.8318(3)	17.4124(8)
β [°]	99.944(3)	108.956(4)
*V* [Å^3^]	1622.47(2)	3025.7(2)
*d*(Pb_1_–Pb_2_) [Å]	3.9167(4)	3.931(6)
*d*(Pb_1_–Pb_3_) [Å]		5.962(9)

The crystal structures of the DJ phases are shown in Figure 3. They confirm the synthesis of layered lead–bromide phases. “k”-C_4_Ferov consists of chains of face-sharing lead–bromide octahedra. The 1D chains are arranged next to each other, giving the phase a quasi-layered structure. The ferrocene ligands connect the chains in a zigzag pattern (Figure 3a). As the ferrocene ligands lie almost parallel to the inorganic layer, the distance between the inorganic layers is very small, which could already be seen in the PXRD data (Figure 1a) from the calculated interplanar distance. The octahedral network of “e”-C_4_Ferov exhibits face-sharing trimers (Figure 3c,d). The trimers are then connected to each other by corner-sharing bromide ions to form a 2D inorganic network. The ferrocene ligands connect the inorganic layers. There are two ferrocene unit positions in the crystal lattice, which are rotated by almost 90° relative to each other. As both phases exhibit a bridging ferrocene spacer, though a different octahedral connection than the typical corner-sharing arrangement of DJ phases, we named the “k” and “e” phase quasi-DJ. This nomenclature also applies to all other phases, showing different octahedral connections but similarities with DJ or RP phases and to the quasi-RP in our previous work.^24^

**Figure 3:**
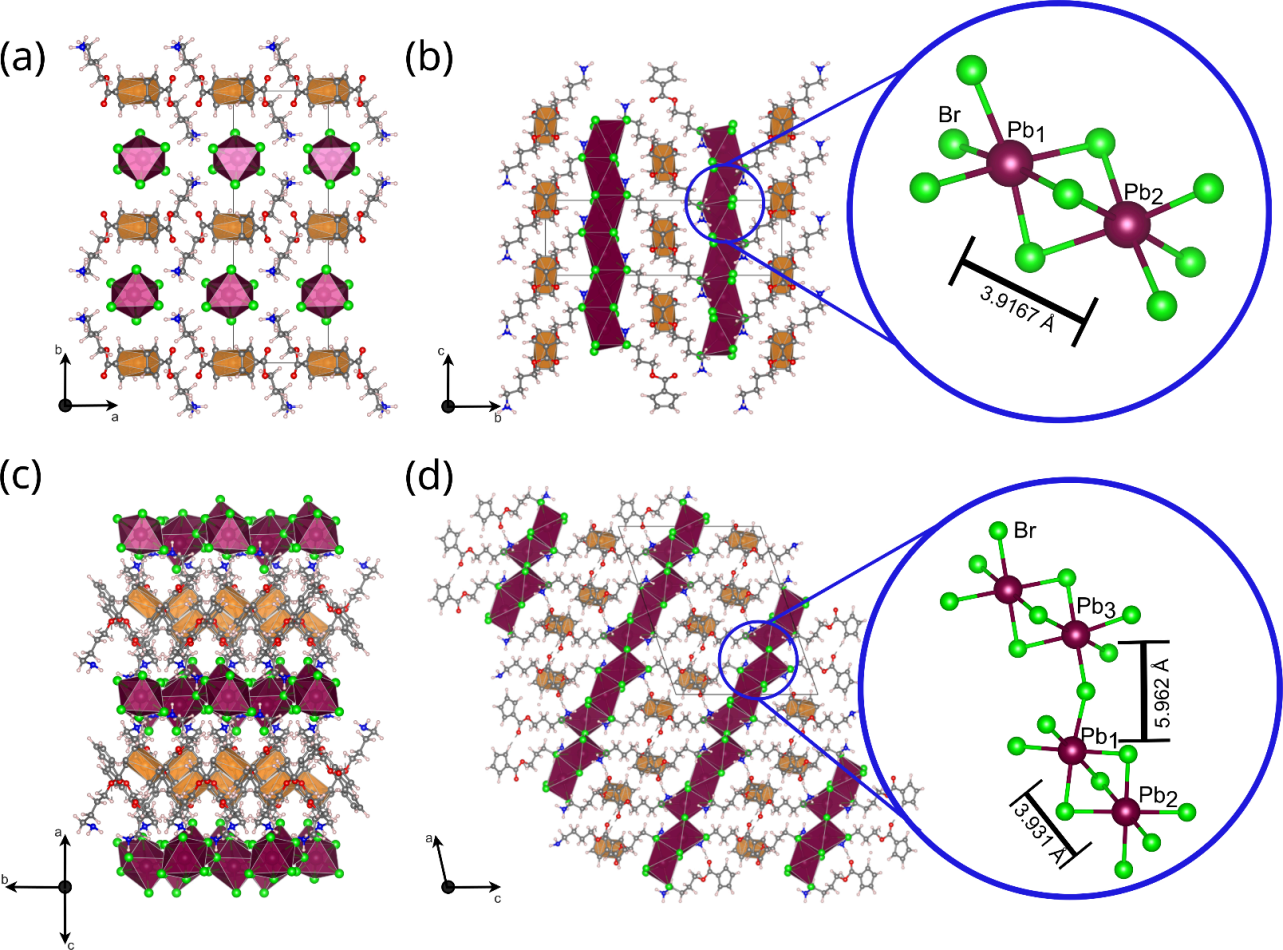
Crystal structure with view along different axis’ and their octahedral connectivity and Pb–Pb distances of (a, b) “k”-C_4_Ferov with face-sharing 1D octahedral chains and (c, d) “e”-C_4_Ferov with a 2D network of face- and corner-sharing octahedra. Color code: Pb octahedra in purple with green bromide anions and the ferrocene units in orange, carbon atoms: gray, nitrogen: blue, hydrogen: white.

A comparison of the octahedral connection of both quasi-DJ phases confirms kinetic and thermodynamic control over the synthesis. The kinetic “k”-C_4_Ferov phase contains only face-sharing octahedra, which are unfavorable for ionic structures, as the lead ions in the center of the octahedra are closer to each other and exhibit stronger electrostatic repulsion.^11^ This effect can also be seen in the distortion of the octahedra in Figure 3. Meanwhile, the “e”-C_4_Ferov phase consists of only small face-sharing moieties, trimers, connected to each other by corners, a more favorable arrangement, giving the outer lead ions the possibility to shift to the outside facet of the trimers to avoid repulsion. The avoidance of the Pb ions is also evident in the increased Pb–Pb distance of 3.931 Å, compared to 3.916 Å in the face-sharing chain (Figure 3b,d and Table 1).

Because DJs and RPs have different compositions, since RP phases consist of corner-sharing octahedra with twice as many spacer cations in the organic layer, we intentionally changed the spacer-to-lead ratio to 2:1 in the slow antisolvent synthesis, hoping this would shift the equilibrium. The PXRD of the resulting “s”-C_4_Ferov (“s” corresponds to stoichiometric) is shown in Figure 1a. The crystallinity is lower due to expected strong stacking disorders, typical for RP phases with large, flexible molecules.^24^ They can be deduced from the low number of reflections, asymmetric reflections (peak broadening to higher scattering angles), and direction-dependent reflection widths in the PXRD (Figure 1a).^44^ Due to the extremely short lifetime of “s”-C_4_Ferov in the electron beam, 3D ED acquisitions of the phase were not possible. We were also not able to index and observe diffuse scattering based on individual 2D diffraction patterns since the features were hidden due to the preferred crystal orientation on the TEM grid (stacking axis parallel to the electron beam at a stage tilt of 0°). Nevertheless, the observed pattern correlates well with an RP phase, with an interlayer spacing of *d* = 21.5 Å. A PXRD comparison of our RP and the literature known can be found in the SI (Figure S15) and agrees with the successful synthesis of an RP phase.

As we have now proved the presence of two quasi-DJ phases and one RP phase, we successfully synthesized DJ, quasi-DJ, and RP phases from the same precursor molecule. Even though the RP and quasi-DJ phases do not show similar octahedral arrangements, this marks a first step toward the possibility of comparing RP and DJ phases directly. Furthermore, ferrovskites from other works showed 1D RP and 2D quasi-RP phases similar to our quasi-DJ phase octahedral arrangements.^10,24^ This means that it is generally possible to achieve similar octahedral layers to our quasi-DJ phases and also for quasi-RP phases.

### 3.3 Structure–Property Correlations of C_4_Ferovs

Through our synthesis efforts, we delivered three different layered hybrid perovskites, all of which contain the same linker species Fc­(C_4_Br)_2_ and different octahedral arrangements. Therefore, it is interesting to compare the optical properties with each other. Figure 4a,b shows the data obtained from UV–vis measurements. In layered materials, such as 2D hybrid perovskites, the electrons are confined, which is expressed by the appearance of an exciton peak, which directly correlates with the band gap.^45^ “k”-C_4_Ferov shows the exciton peak at the highest energy (3.77 eV), followed by “e” (3.48 eV) and “s” (3.08 eV). The band gap decreases in the following order: “k” > “e” > “s”, which is exactly what one expects for the difference in face-sharing > face-corner-sharing > corner-sharing of the octahedra.^46^ The values agree with reports in the literature on typical corner-sharing DJs/RPs.^24,47^ The primary influence on the properties stems from the connectivity of the lead–bromide octahedra and the resulting change in the quantum confinement situation. An increase in the confinement results in an increase in the exciton binding energy and band gap. However, the position of the exciton peak also derives from a combination of two factors. In addition to the quantum confinement mentioned above, dielectric confinement exists. Here, the ferrocene influences the polar surrounding of the perovskite lattice based on the contrast in dielectric constants. It therefore also influences the localization of charge carriers.^45^ The band structure was characterized in further detail using photoelectron spectroscopy (Figure 4b) and confirmed the described findings.

**Figure 4:**
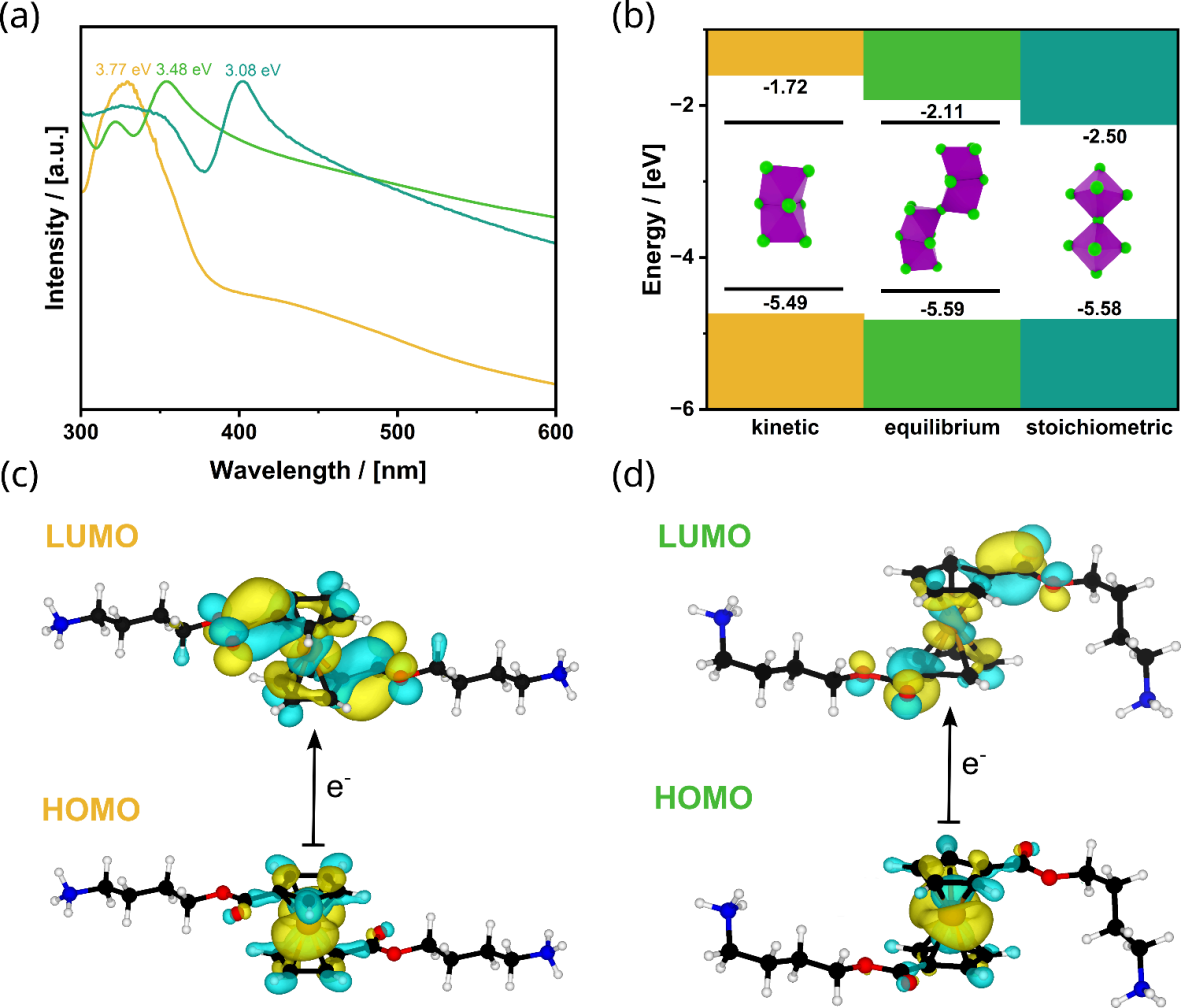
(a) UV–vis spectra of “k”- (yellow), “e”- (green), and “s”- (turquoise) C_4_Ferovs and (b) schematic representation of the band gaps for all C_4_ ferrovskite phases. The results were derived from both photoelectron spectroscopy in air (PESA) (Figures S17 and S18) and the UV–vis data. Primarily, the position of the conduction band varies for all phases. The HOMO and LUMO position of the ferrocene from the calculated data are shown in black in between the VB and CB. 3D isosurface of the HOMO and LUMO orbital of the ferrocene in the “k”- (c) and “e”- (d) phases from DFT calculations.

Ferrocene is a chromophore by itself. The spectrum of the free ferrocene spacer Fc­(C_4_Br)_2_ displays two absorption bands at *E*_1_ ≈ 2.7 eV and *E*_2_ ≈ 3.5 eV. The HOMO–LUMO transition of the d–d type is Laporte forbidden; therefore, the band at *E*_1_ is very weak. However, it is responsible for the orange color of the compound.^26^ The band at *E*_2_ corresponds to a π–π* transition in ferrocenes, and it is in a region similar to the excitonic peak, which was discussed before. The HOMO–LUMO transition of Fc is barely visible in the absorption spectra of all of the C_4_Ferovs phases (Figure 4a). Studies by fluorescence spectroscopy were not possible, unfortunately, because neither the ferrocene nor the perovskite exhibited photoluminescence signals (Figure S16).^24^ For ferrocene, the absence of any luminescence is a well-known case.^48^ Particularly, ferrocene's nature as the electron donor can quench the appearance of photoluminescence of other systems by electron transfers,^49^ which could be the reason for the absence of luminescence signal of the perovskite. This effect is canceled out by oxidation of the ferrocene, as it is then no longer an electron donor.^24^

Based on the obtained structural information, the electronic structure of C_4_Ferovs was studied further by quantum methods. DFT calculations of “k”- and “e”-C_4_Ferovs using the generalized gradient approximation (GGA) by employing the PBEsol XC functional qualitatively agree with the experimentally found values for the band gap; see Supporting Information Figure S19. From the calculated band structures, the projected density of states (PDOS) (Figure S20) one can see that the frontier orbitals of the Fc represent states inside the band gap (Figure 4b). Figure 4c,d shows 3D isosurfaces of the HOMO and LUMO orbital of the linker molecule in the “k”- and “e”-C_4_Ferovs. The orbital coefficients of the HOMO are situated at the Fc unit, whereas there is charge density at the ester functionalities for the LUMO. These ester functionalities are near the lead halide layer, which makes an overlap and thus a delocalization through the organic layer more likely and the material a perfect candidate for future studies on electronic communication. Additionally, in the photoelectron spectra, one can see an energy increase of the HOMO of the free Fc derivative, which is below the valence band edge, compared to the calculations of the Fc integrated into the hybrid perovskite (Figures S18 and S20), where it is above. Even though the absolute values cannot be compared due to the red shift of the GGA values, a relative comparison of the bands and orbitals to each other is possible. The small shift further underlines our suppositions that there is electronic communication between the electronic system of the ferrocene with the crystal orbitals of the semiconductor, at least to a certain degree.

### 3.4 C_*n*_Ferovs—Changing the Spacer Length

The distance between the lead halogenide layers and the Fc, respectively, and the ester functionalities, depends on the alkyl spacer length in Fc­(C_*n*_Br)_2_. It was mentioned in Section 2.1 that spacer molecules for *n* varying from 3 to 6 are available, which are now used to prepare the hybrid perovskites using the same antisolvent method. PXRD was employed to assess the resulting solids (Figure 1c). The interlayer distance *d* is plotted in Figure 1d for the series of described samples. The sample “k”-C_3_Ferov seems to crystallize analogous to the kinetic quasi-DJ shown in Figure 3a,b for “k”-C_4_Ferovs. Surprisingly, the interlayer distance of “k”-C_5,6_Ferov is much larger (*d* > 19 Å), and the PXRD pattern is consistent with typical RPs in the literature.^24,46^ Other than expected, the Fc­(C_*n*_Br)_2_ linkers (*n* = 5,6) have not delivered DJs or quasi-DJs despite the two ammonium groups present. Therefore, we tested whether a slower reaction kinetics (“e” samples) changes the situation. Figure 1d illustrates the correlation of *d* with *n* of the “e”-C_*n*_Ferovs derived from PXRD data, which are shown in the Supporting Information. Now, that there is more time for the linker molecules to adopt a more favorable conformation, the value of *d* and the PXRD pattern of samples with *n* = 5,6 indicate that quasi-DJs resembling the structure of “e”-C_4_Ferov (Figure 3c,d) were formed. “e”-C_3_Ferov (*d* = 20.34 Å) is an outlier and, surprisingly, has transitioned to an RP. The shorter chain length might prohibit the formation of “e”-C_4_Ferov-like phases as the spacer molecules are already stretched out in it. This energetically unfavorable conformation of the ferrocene molecule leads to the preferential formation of an RP phase.

Investigation of the optical properties of the “k”- and “e”-C_*n*_Ferovs follows the trend of the C_4_Ferovs, and the band gap decreases in the following order: “k” > “e” > “s”. The “k”-C_3_ shows optical properties similar to those of “k”-C_4_, which agrees with their structural similarities. In the same manner, the “k”-C_5_- and C_6_-Ferovs exhibit an RP-like exciton peak. The “e”-C_*n*_Ferovs also show properties correlated with their categorization into “e”/“k”-quasi-DJ or RP phases (Figure 5 and S22).

**Figure 5:**
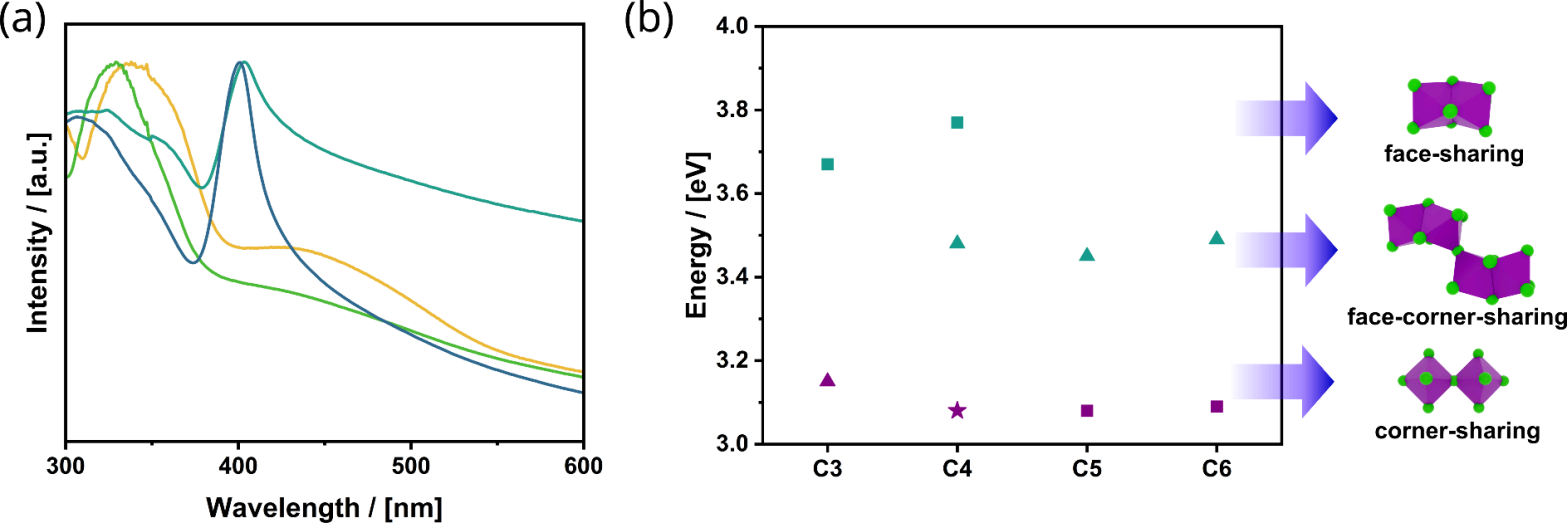
(a) UV–vis spectra of the “k”-C_*n*_Ferovs C_3_ in yellow, C_4_ in green, C_5_ in turquoise, and C_6_ in blue. (b) Exciton peak maximum position of the “k”- (squares), “e”-(triangles) C_*n*_Ferovs, and the “s”-C_4_Ferovs RP phase (star) with quasi-DJ phases in turquoise and RP in purple.

## 4 Conclusions

Understanding the structure-directing effect of ligands in hybrid materials is crucial for tailoring them to specific applications. Often, the integration of the ligands is a challenge. Here, for the first time, quasi-DJ ferrocene hybrid perovskites were synthesized with a bridging diammonium spacer. Even though it is challenging to arrange large, rigid units such as ferrocene in the small space provided by DJ phases, with an alkyl chain length of four carbon atoms, necessary flexibility to form quasi-DJ phases, DJ phases with a changed octahedral connectivity, was provided. In addition, the structure-directing effect of these large organic units was used to synthesize three different ferrovskite phases from the C_4_-spacer by varying the reaction time and stoichiometry, demonstrating both thermodynamic and kinetic control of the synthesis, as well as the influence of the stoichiometry on ferrovskite formation. The kinetically and thermodynamically/equilibrium-controlled syntheses led to two quasi-DJ phases and an RP phase. They all show layered structures with different octahedral networks: 1D face sharing for the kinetic quasi-DJ phase, 2D face-corner sharing for the thermodynamic quasi-DJ phase, and corner sharing for the RP phase. Those three phases resemble the octahedral connection of the inorganic manganate perovskite minerals with different-sized A cations. However, instead of exchanging the cation, we enabled the option to vary the structure by changing the synthesis conditions. The structures of the fine crystalline powder samples were determined with a combination of 3D electron diffraction, DFT optimizations, and Rietveld refinements. Their optical and electronic properties differ depending on the dimension and connectivity of the octahedral network, with a decreasing band gap from face- to corner-sharing octahedra. This enables the possibility to use one precursor molecule to address different properties demanded for applications and clears the path for the possibility of a direct comparison of those materials, even quasi-RPs and quasi-DJs, as they consist of the same educts. This work not only underscores the potential of ferrocene-based hybrid perovskites but also opens new avenues for the design of advanced 2D hybrid perovskite materials with tailored functionalities for various applications.

## Supplementary Material





## Notes

^1^H and ^13^C NMR spectra for all compounds; reaction scheme for the spacer synthesis; and PXRD for all “e”-ferrovskites and spacers, IR, fluorescence, PESA, and UV–vis spectra, SEM micrographs, as well as details regarding 3D ED results and DFT calculations (PDF)
Crystal structure information for the “k”- and “e”-quasi-DJ phases added separately (ZIP)
