# Supplementary material for: Accessing Multiple Phases via Thermodynamic or Kinetic Pathways: The Impact of Bivalent Ferrocene Spacers on 2D Hybrid Perovskite Formation
Source: ACS Appl Mater Interfaces. 2025 Oct 24;17(44):60997–1006. doi: 10.1021/acsami.5c14485 (PMC12598695; doi:10.1021/acsami.5c14485)
Supplement: Supplementary file 1 [file am5c14485_si_001.pdf]

Supporting Information

Accessing Multiple Phases via Thermodynamic or Kinetic Pathways: The Impact of Bivalent Ferrocene-Spacers on 2D Hybrid Perovskite Formation

Melina Dahlke[1,2], Yaşar Krysiak[1], Marvin Treger[2,3], Carolin König[2,3], Sebastian Polarz[1,2]**

[1]Institute of Inorganic Chemistry, Leibniz University of Hannover, Callinstraße 9, 30167 Hannover, Germany.

[2]Cluster of Excellence PhoenixD, Leibniz University of Hannover, Welfengarten 1A, 30167 Hannover, Germany.

[3]Insitute of Physical and Electrochemistry, Leibniz University of Hannover, Callinstraße 3A, 30167 Hannover, Germany.

e-mail: sebastian.polarz@aca.uni-hannover.de; yasar.krysiak@aca.uni-hannover.de

Table of Contents

- Figure S1: Reaction scheme of the organic spacer synthesis.
- Figure S2: ^{1}H- and ^{13}C -NMR of the $Fc(C_6Br)_2$ spacer cation.
- Figure S3: ^{1}H- and ^{13}C -NMR of the $Fc(C_5Br)_2$ spacer cation.
- Figure S4: ^{1}H- and ^{13}C -NMR of the $Fc(C_4Br)_2$ spacer cation.
- Figure S5: ^{1}H- and ^{13}C -NMR of the $Fc(C_3Br)_2$ spacer cation.
- Figure S6: Powder X-ray diffractograms and UV-Vis spectra of all spacer cations.
- Figure S7: IR spectra of the ferrocene perovskites.
- Figure S8: SEM micrographs of the fast crystallized C_3-C_6 'k'-C_nFerovs particles.
- Figure S9: SEM micrographs of the 'k'-, 'e'- and 's'-C_4Ferovs.
- Figure S10: Scheme of the synthesis methods of the hybrid perovskites.
- Table S1: Relevant parameters of the 3D electron diffraction measurement, crystallographic data and refinement results of 'k'-C4Ferov and 'e'-C_4Ferov.
- Figure S11: Crystallographic sections 0kl, h0l, and hk0 of 'k'-C_4Ferov and 'e'-C_4Ferov.
- Figure S12: Comparison of the XRD patterns of the experimental and DFT optimized structure data.
- Table S2: Comparison of the experimental lattice parameters and the DFT results.
- Figure S13: Plane wave basis set convergence of the kinetic C_4 ferrovskite phase.
- Figure S14: Plane wave basis set convergence of the thermodynamic C_4 ferrovskite phase.
- Figure S15: Comparison of the PXRD of the 's'-RP phase (C4Ferov) and the literature RP phases.
- Figure S16: Fluorescence spectra of the C_4 Ferrovsites and spacer molecules.
- Figure S17: PESA measurements and band gap of the kinetic C_3-C_6.
- Figure S18: PESA of the 'e'- and 's'-C_4 ferrovskite phases.
- Figure S19: Comparison of the calculated absorption coefficients of the C_4 DJ ferrovskite phases.
- Figure S20: Generalized gradient approximations for both C_4 DJ phases.
- Figure S21: PXRDs of the 'e'-C_nFerovs.
- Figure S22: UV-Vis spectra of the ferrovskites with chain lengths C_3-C_6 of the 'e'-C_nFerovs phases.

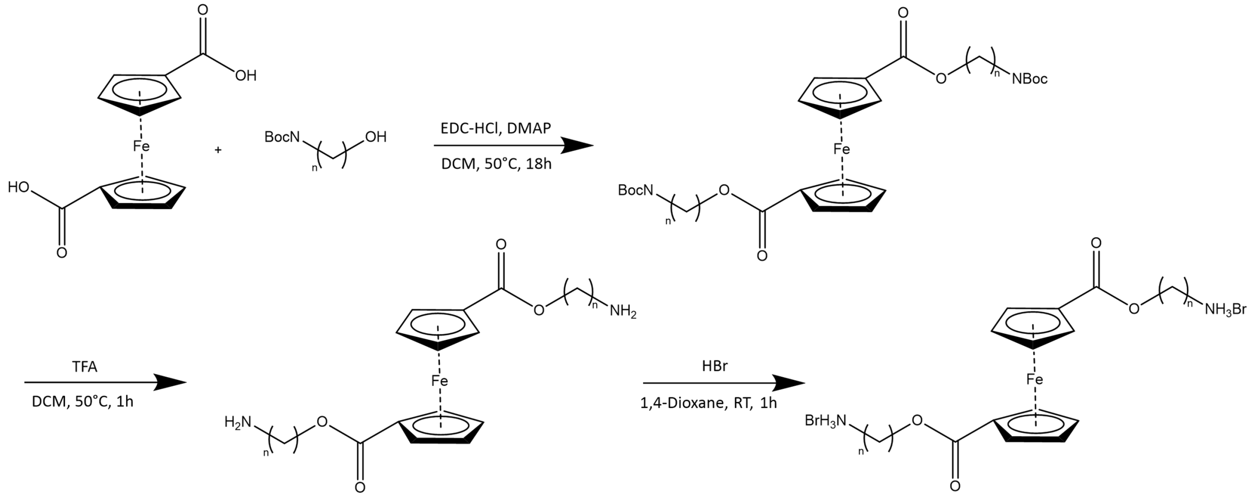

Figure S1. Reaction scheme of the synthesis of the divalent organic spacer cation $Fc(C_nBr)_2$ with n = 3,4,5,6. Starting from the 1,1'-ferrocene dicarboxylic acid, first step: Esterification, followed by deprotection of the BOC-group and brominating.

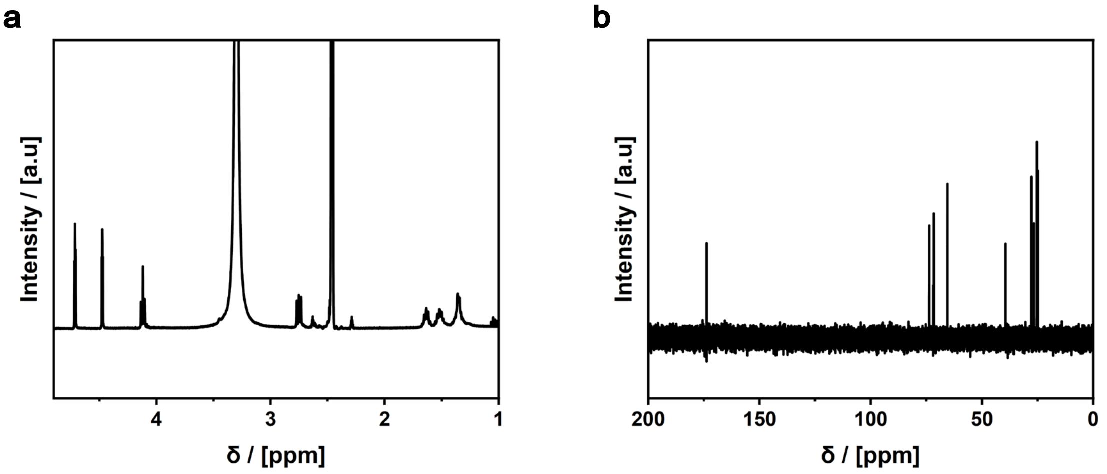

Figure S2. a) ^{1}H-NMR and b) ^{13}C-NMR of the $Fc(C_6Br)_2$ spacer cation. The evaluation of the spectra can be found in the Experimental Section. ^{1}H-NMR (400 MHz, DMSO-d6), δ (ppm)= 4.71 (t, 4H, CH), 4.48 (t, 4H, CH), 4.12 (t, 4H, CH_2), 2.75 (t, 4H, CH_2), 1.64 (m, 4H, CH_2), 1.52 (m, 4H, CH_2), 1.35 (m, 8H, CH_2), 2.46 (t, DMSO), 3.30 (s, H_2O), 1.05 (m, ethanol). ^{13}C-NMR (400 MHz, D_2O), δ (ppm)= 173.78 (2C, O=C-O), 73.74 (4C, CH-Cp), 71.89 (2C, CH-Cp), 71.67 (4C, CH-Cp), 65.54 (2C, CH_2-O), 39.45 (2C, CH_2-NH_3), 27.77 (2C, CH_2), 26.68 (2C, CH_2), 25.32 (2C, CH_2), 24.81 (2C, CH_2).

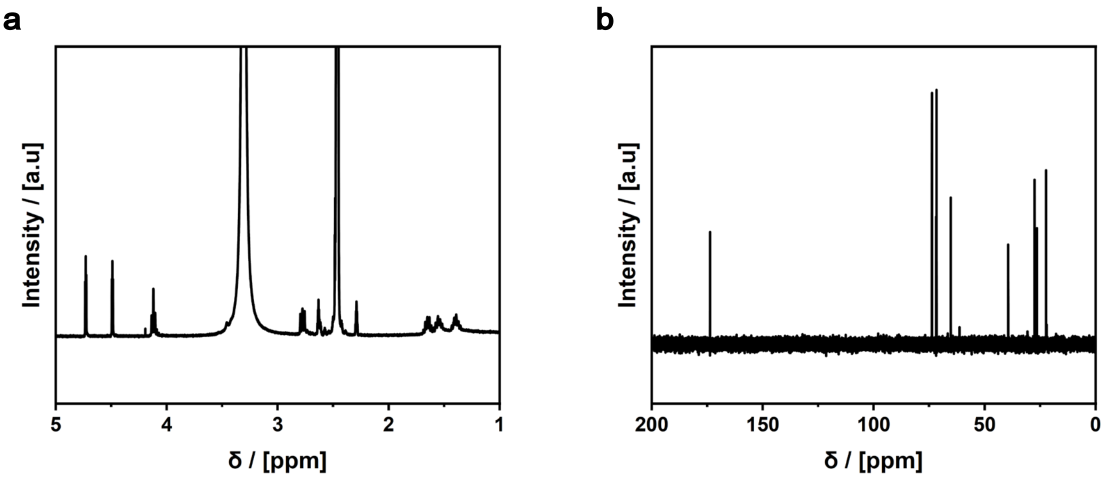

Figure S3. a) ^{1}H-NMR of $Fc(C_5Br)_2$ ligand. b) ^{13}C-NMR of $Fc(C_5Br)_2$ ligand. The evaluation of the spectra can be found in the Experimental Section. ^{1}H-NMR (400 MHz, DMSO-d6), δ (ppm)= 4.73 (t, 4H, CH), 4.49 (t, 4H, CH), 4.12 (t, 4H, CH_2), 2.78 (t, 4H, CH_2), 1.65 (m, 4H, CH_2), 1.56 (m, 4H, CH_2), 1.39 (m, 4H, CH_2), 2.46 (t, DMSO), 3.30 (s, H_2O). ^{13}C-NMR (400 MHz, D_2O), δ (ppm)= 173.14 (2C, O=C-O), 73.75 (4C, CH-Cp), 71.85 (2C, CH-Cp), 71.67 (4C, CH-Cp), 65.25 (2C, CH_2-O), 39.38 (2C, CH_2-NH_3), 27.52 (2C, CH_2), 26.47 (2C, CH_2), 22.35 (2C, CH_2).

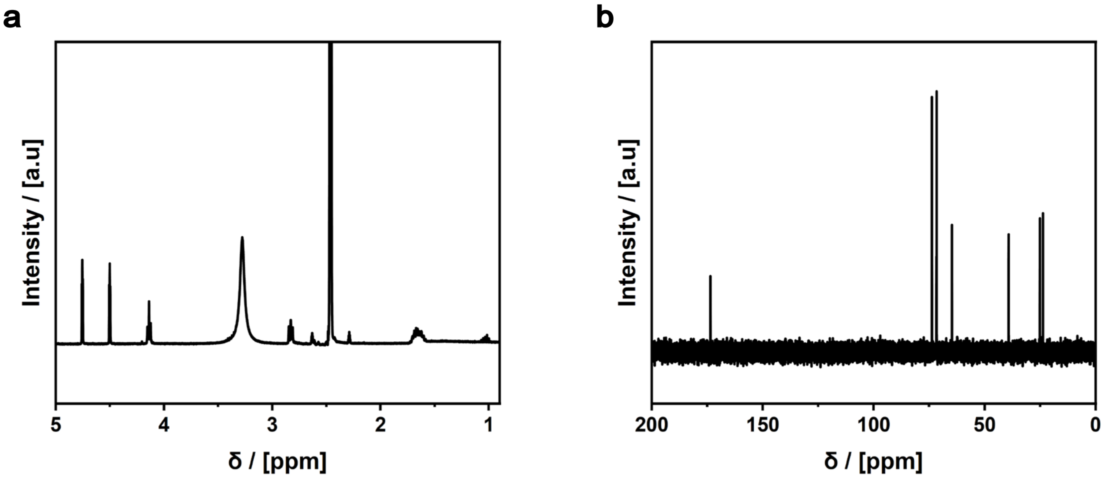

Figure S4. a) ^{1}H-NMR of $Fc(C_4Br)_2$ ligand. b) ^{13}C-NMR of $Fc(C_4Br)_2$ ligand. The evaluation of the spectra can be found in the Experimental Section. ^{1}H-NMR (400 MHz, DMSO-d6), δ (ppm)=

4.75 (t, 4H, CH), 4.50 (t, 4H, CH), 4.14 (t, 4H, CH_2), 2.83 (t, 4H, CH_2), 1.66 (m, 8H, CH_2), 2.46 (t, DMSO), 3.28 (s, H_2O), 1.03 (m, ethanol). ^{13}C-NMR (400 MHz, D_2O), δ (ppm)= 173.54 (2C, O=C-O), 73.77 (4C, CH-Cp), 71.75 (2C, CH-Cp), 71.65 (4C, CH-Cp), 64.74 (2C, CH_2-O), 39.19 (2C, CH_2-NH_3), 25.10 (2C, CH_2), 23.22 (2C, CH_2).

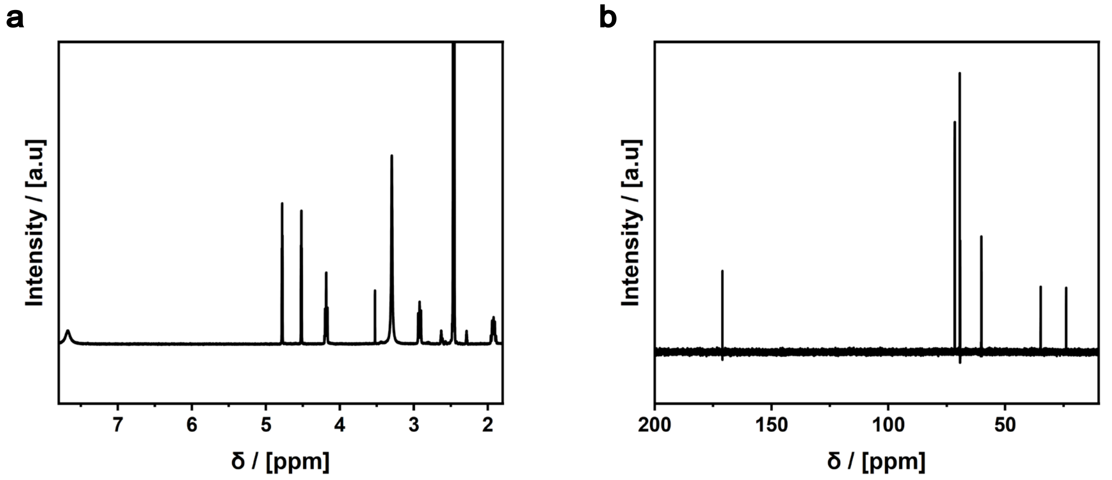

Figure S5. a) ^{1}H-NMR of $Fc(C_3Br)_2$ ligand. b) ^{13}C-NMR of $Fc(C_3Br)_2$ ligand. The evaluation of the spectra can be found in the Experimental Section. ^{1}H-NMR (400 MHz, DMSO-d6), δ (ppm)= 7.68 (s, 6H, NH_3), 4.78 (t, 4H, CH), 4.52 (t, 4H, CH), 4.18 (t, 4H, CH_2), 2.92 (t, 4H, CH_2), 1.92 (m, 4H, CH_2), 2.46 (t, DMSO), 3.30 (s, H_2O), 3.53 (s, 1,4-dioxane). ^{13}C-NMR (400 MHz, D_2O), δ (ppm)= 172.82 (2C, O=C-O), 71.58 (4C, CH-Cp), 69.43 (4C, CH-Cp), 69.29 (2C, CH-Cp), 60.14 (2C, CH_2-O), 34.79 (2C, CH_2-NH_3), 23.88 (2C, CH_2).

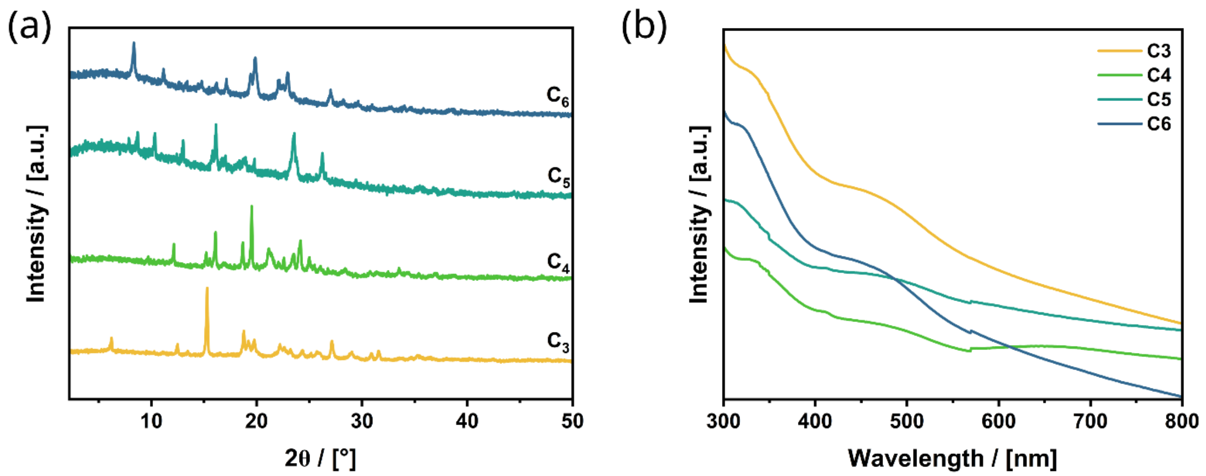

Figure S6. a) Powder X-ray diffractograms (PXRD) of the $Fc(C_nBr)_2$ ligands with the chain lengths n = 3-6. b) UV-Vis absorption spectra as dispersion in toluene of the $Fc(C_nBr)_2$ ligands with the chain lengths n = 3-6. C_3 = gold, C_4 = green, C_5 = turquoise, C_6 = blue. All molecules show crystalline reflexes and different pattern than in the perovskites. All UV-Vis spectra are similar to each other. Therefore, the influence of the chain length on the optical properties of the molecules is negligible.

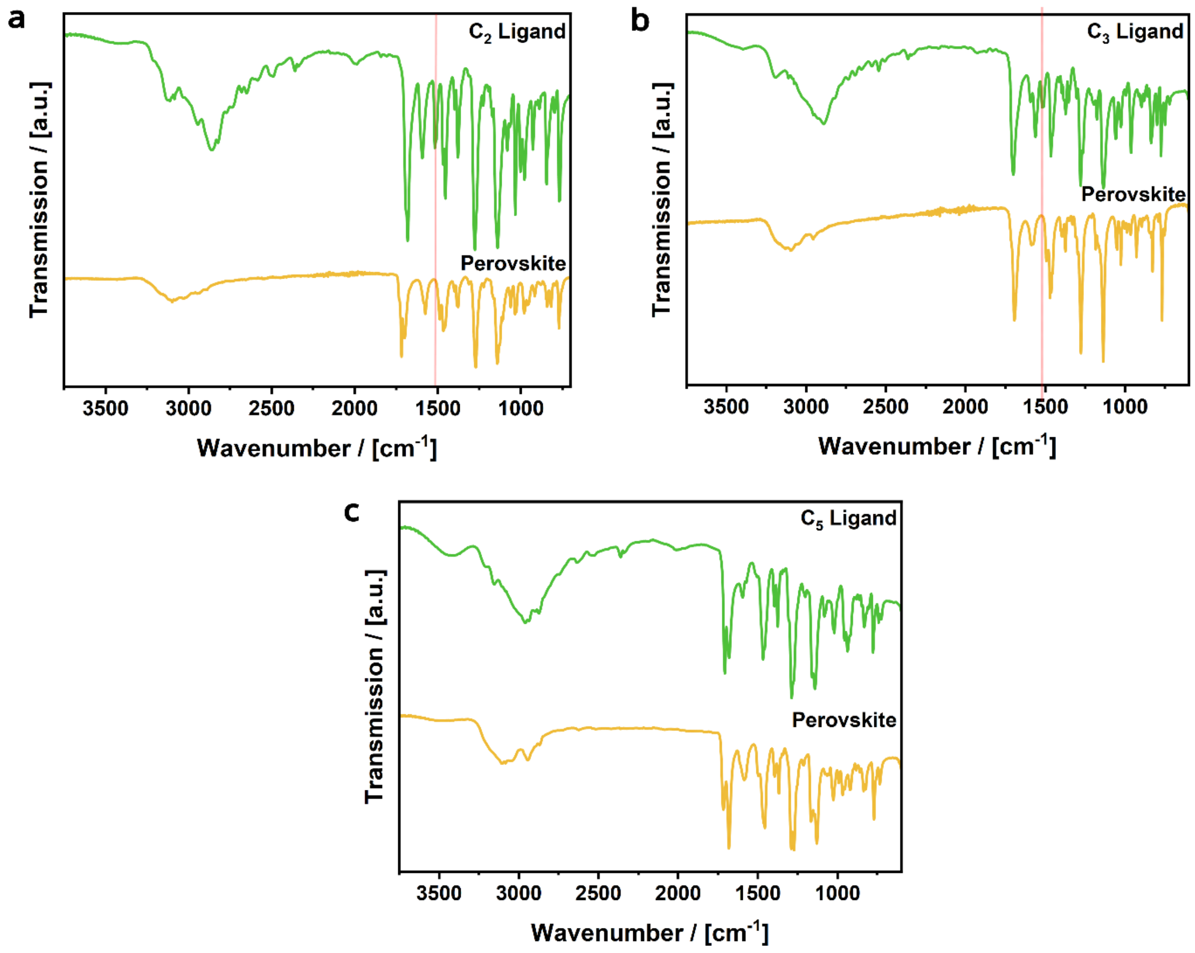

Figure S7. IR spectra of the ligand and perovskite of a) 'k'-C_3, b) 'e'-C_4Ferov and c) 'k'-C_5. The red line indicates the position of the ammonium vibration band which vanishes due to the H-bond with the inorganic layer in the perovskites.

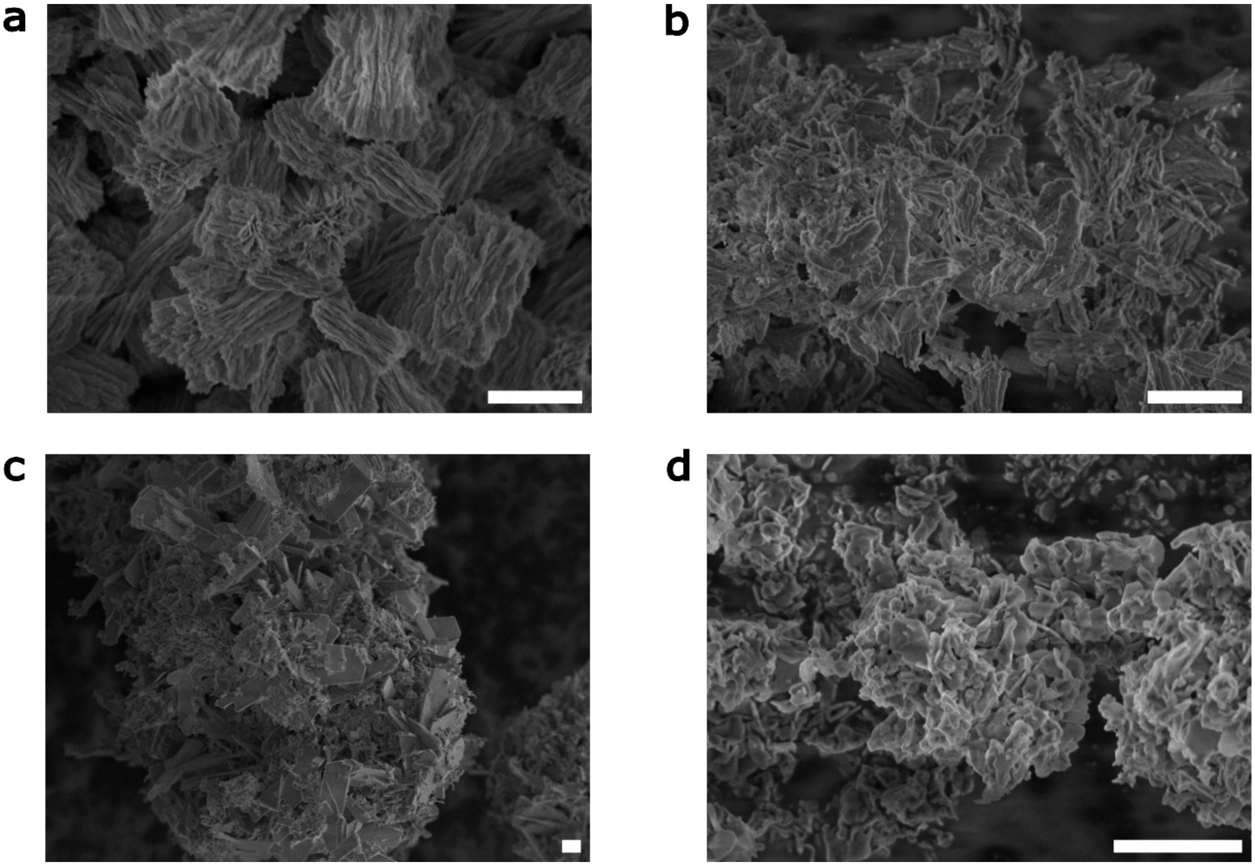

Figure S8. Scanning electron microscope micrographs of the a-d) C_3-C_6 fast crystallized 'k'-C_nFerovs particles. Scalebar: 2 µm. All phases show plate like particles indicating the successful layered material synthesis.

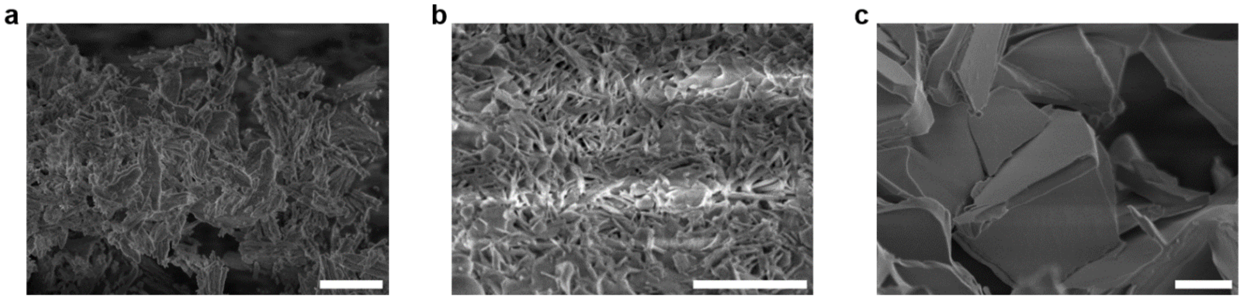

Figure S9. Scanning electron microscope micrographs of the a-c) 'k'-, 'e'- and 's'-C_4Ferovs. Scalebar: 2 µm. All phases show plate like particles indicating the successful layered material synthesis.

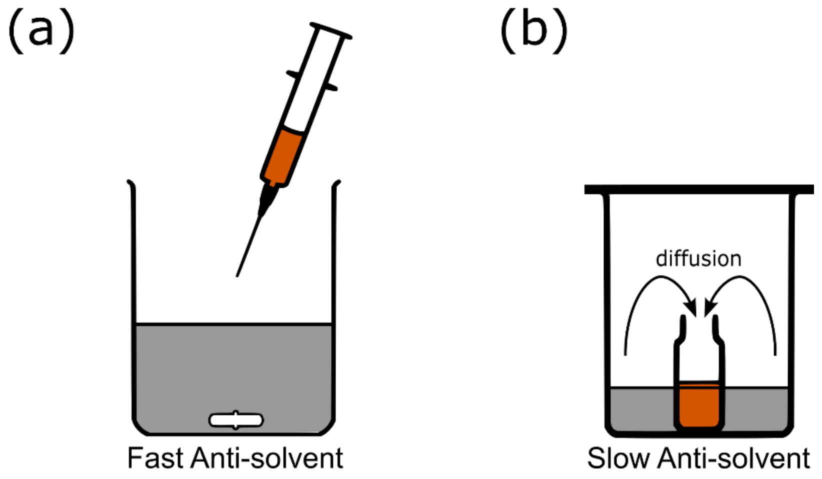

Figure S10. Scheme of the synthesis methods of the hybrid perovskites. a) fast anti-solvent and b) slow diffusion anti-solvent synthesis. The precursor solution is marked in orange and the anti-solvent in grey.

Table S1. Relevant parameters of the 3D electron diffraction measurement, crystallographic data and refinement results of 'k'-C_4Ferov and 'e'-C_4Ferov.

	'k'-C_4Ferov	'e'-C_4Ferov
**Crystal data**		
Crystal system, space group	monoclinic, *P* $2_1/c$	monoclinic, *P* $2_1/n$
a, *b*, *c* (Å)	8.634(4), 24.31(2), 7.840(4)	21.854(7), 8.425(2), 17.483(9)
β (deg.)	99.94(4)	109.24(3)
V (Å³)	1621(2)	3039(2)
Empirical formula	$Pb_2Br_6FeC_{20}N_2O_4H_{30}$	$Pb_3Br_{10}Fe_2C_{40}N_4O_8H_{60}$
Z	2	2
**Data collection**		
Radiation type	electrons, 120 kV	
Wavelength (Å)	0.033492	
Temperature (K)	293	
Integration semi-angle (deg.)	0.4694	0.532
α_{min}, α_{max} (deg.)	-60, +59	-50, +50
No. of frames	119	94
Total accumulated dose (e^-/Å^2)	8.8	10.2
Resolution (Å^{-1})	1	1
Completeness (%)	81	75
**Structure solution**		
R_F (%)	30.7	30.5
**Kinematical structure refinement**		
No. of used reflections (obs/all)	545/1382	884/2389
R_1^{obs}/R_1^{all}, wR_1^{obs}/wR_1^{all} (%)	19.7/41.3, 26.3/34.5	23.0/38.8, 29.1/32.2
goodness of fit (obs/all)	2.54/1.76	3.57/2.42
No. of refined parameters	59	112
$\sigma[\Delta V(\mathbf{r})]$ (e/Å)	0.429	0.236
min[$\Delta V(\mathbf{r})$], max[$\Delta V(\mathbf{r})$] (e/Å)	-1.93, +2.16	-1.93, +2.39

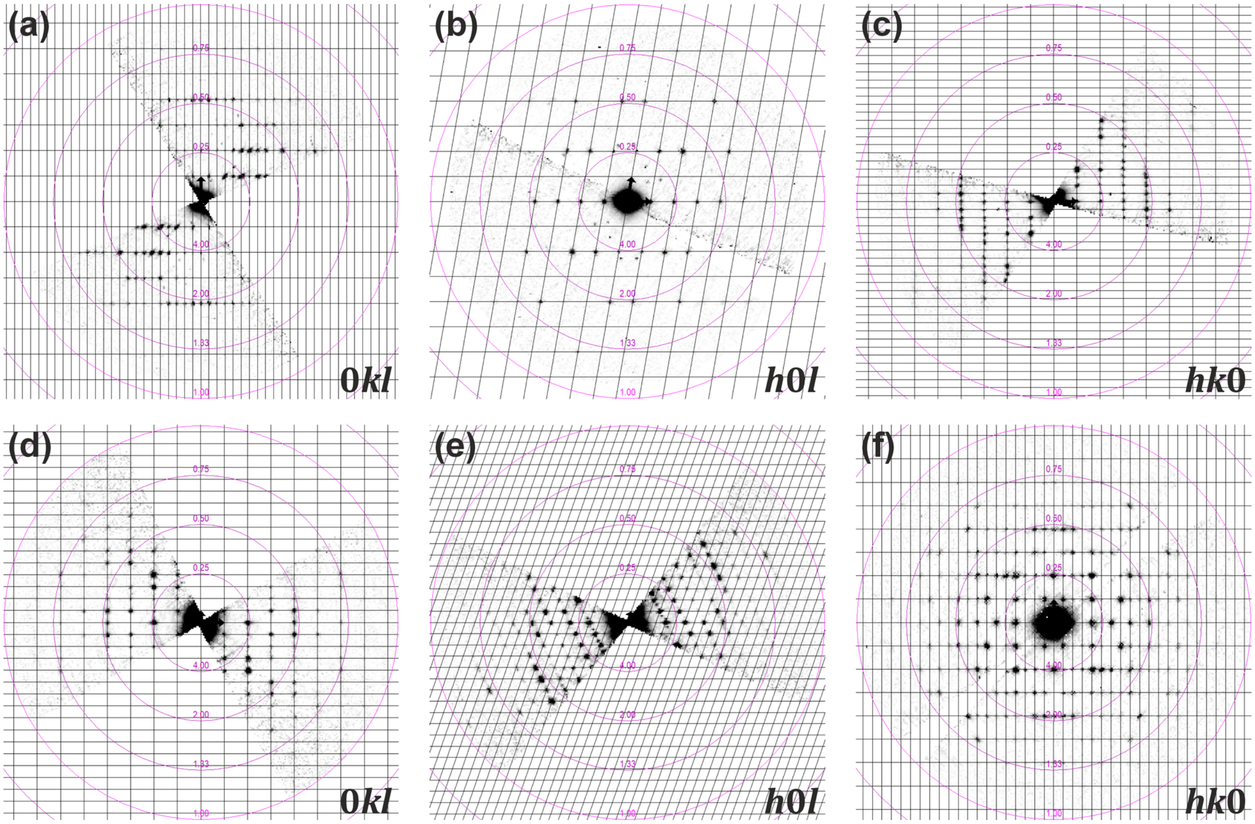

Figure S11. Crystallographic sections 0kl, h0l, and hk0 of 'k'-C_4Ferov (a-c) and 'e'-C_4Ferov (d-f), reconstructed from the individual measured frames recorded using 3D electron diffraction.

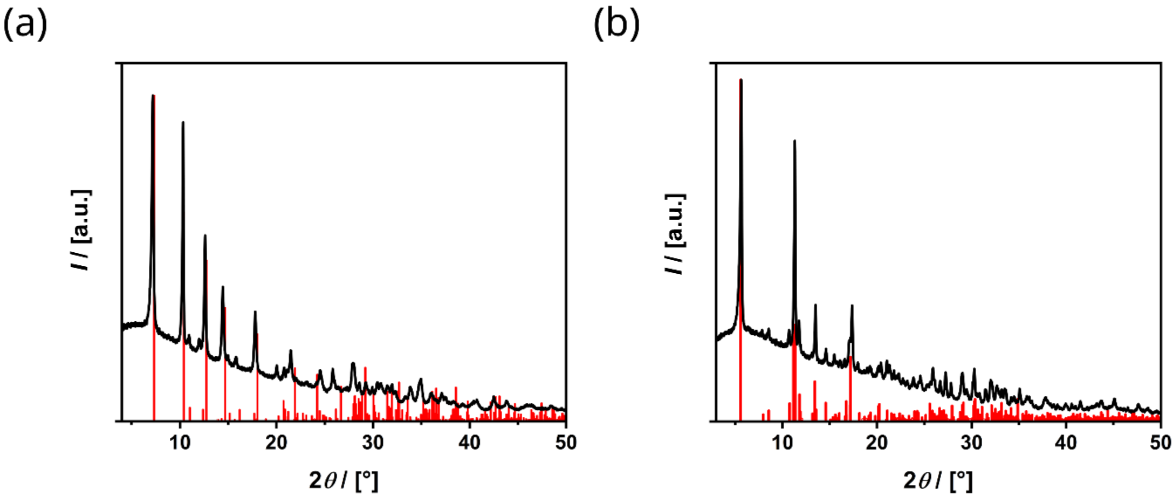

Figure S12. Comparison of the XRD patterns with experimental data (black) and data of the DFT optimized structure (red) of the a) 'k'- and b) 'e'-C_4 ferrovskite phase. Bot show very good

agreement. Smaller differences of the reflections derive from the DFT calculations which correspond to structures at 0 K, while the experiment was carried out at room temperature.

Table S2. Comparison of the experimental lattice parameters and the DFT results of a) the 'k'- and b) 'e'-C_4 ferrovskite phase. Both show very small relative errors indicating good agreement of the experimental and calculated structure model.

a)	Parameter	Exp. (Å)	DFT (Å)	Relative error (%)
	a	8.40	8.74	4.03
	b	24.40	24.11	1.16
	c	7.73	7.68	0.63
b)				
	a	21.79	22.06	1.20
	b	8.43	8.42	0.05
	c	17.44	17.56	0.68

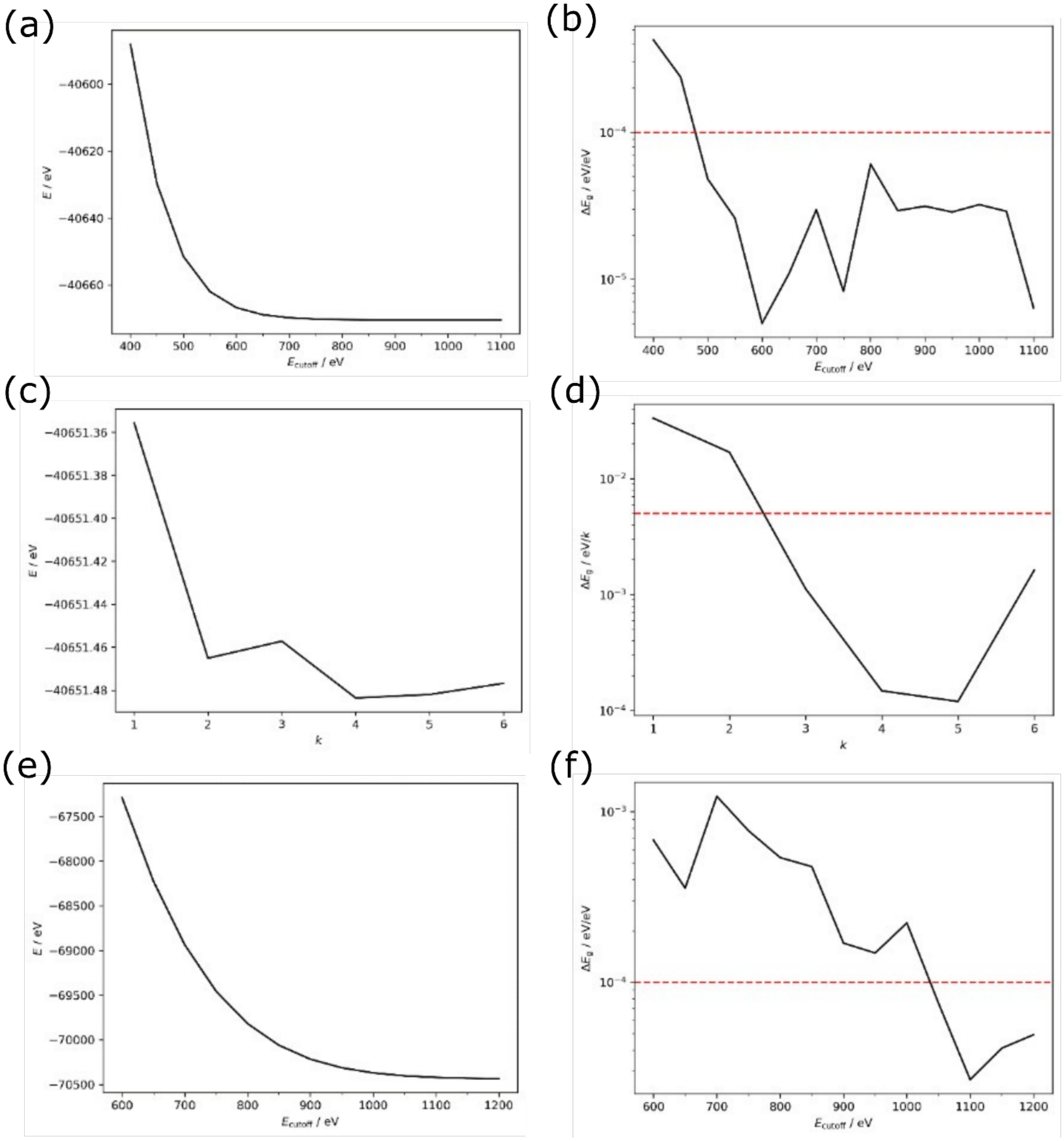

Figure S13. Convergence of the 'k'-C_4 ferrovskite phase of a,b) the plane-wave kinetic energy cutoff with respect to the total energy and the derivative of the band gap energy (threshold 1.0×10^{-4} eV per eV) using ultra-soft pseudopotentials. c,d) the *k* point MP grid with respect to the total energy and the derivative of the band gap energy (threshold 5.0×10^{-3} eV per atom and *k*) using ultra-soft pseudopotentials. e,f) the plane-wave kinetic energy cutoff with respect to the total

energy and the derivative of the band gap energy (threshold 1.0×10^{-4} eV per eV) using norm-conserving pseudopotentials.

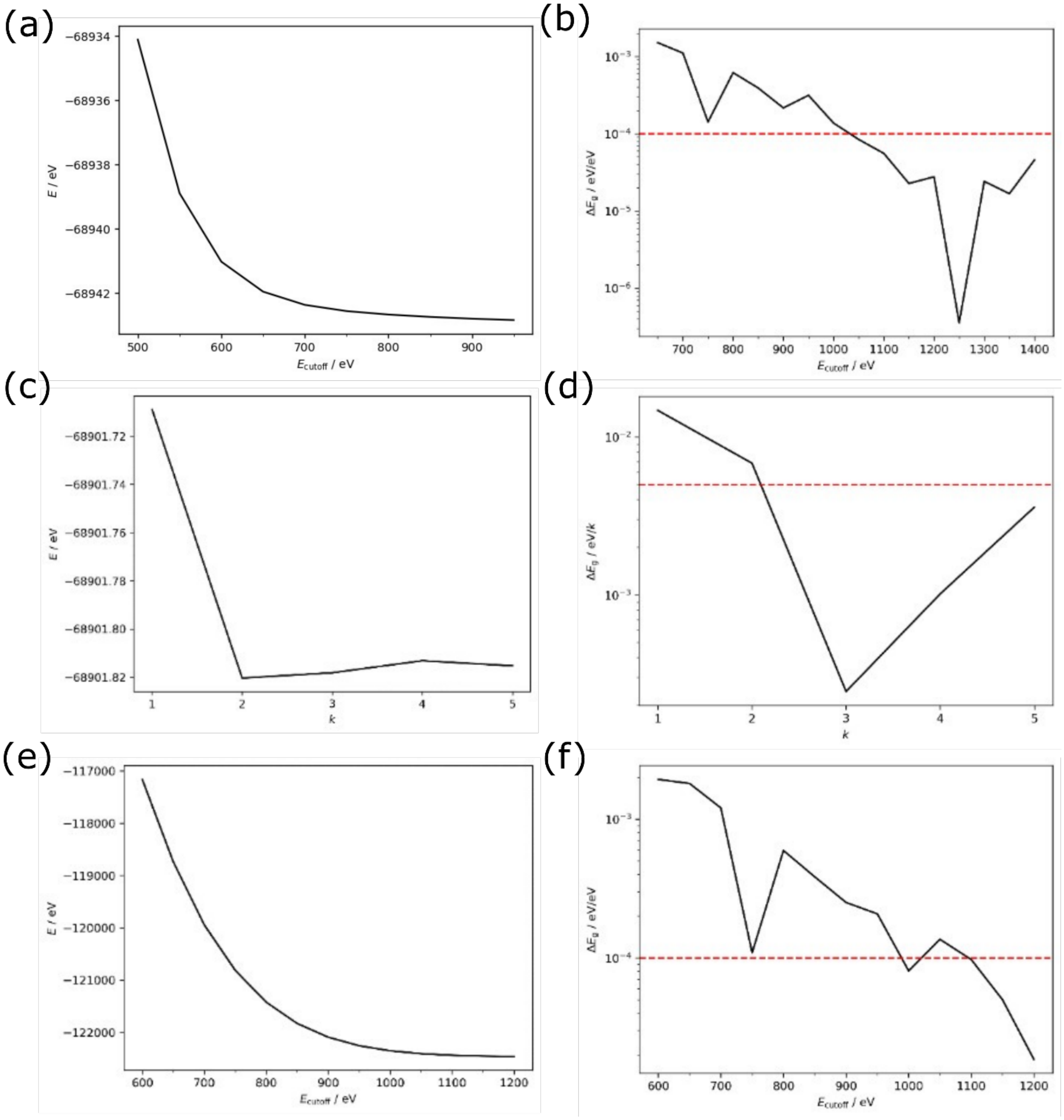

Figure S14. Convergence of the 'e'-C_4 ferrovskite phase of a,b) the plane-wave kinetic energy cutoff with respect to the total energy and the derivative of the band gap energy (threshold 1.0×10^{-4} eV per eV) using ultra-soft pseudopotentials. c,d) the k point MP grid with respect to

the total energy and the derivative of the band gap energy (threshold 5.0 × 10^{-3} eV per atom and *k*) using ultra-soft pseudopotentials. e,f) the plane-wave kinetic energy cutoff with respect to the total energy and the derivative of the band gap energy (threshold 1.0 × 10^{-4} eV per eV) using norm-conserving pseudopotentials.

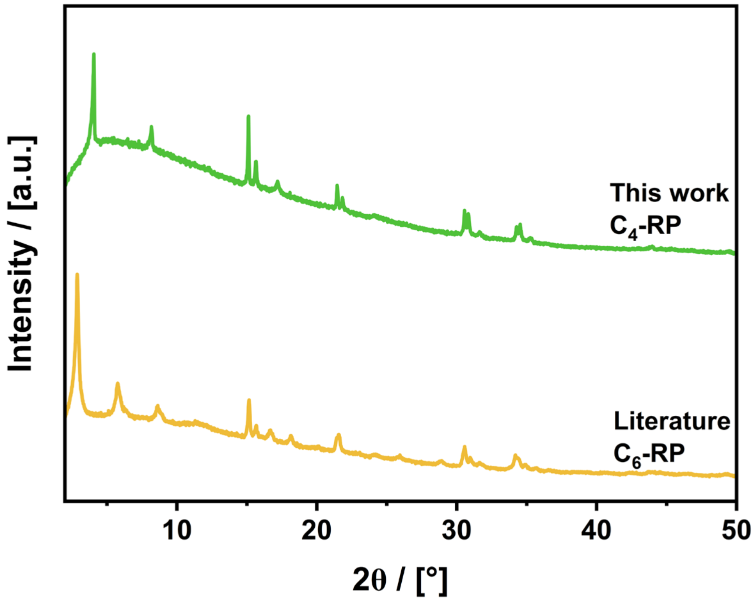

Figure S15. Comparison of the PXRD of the 's'-RP phase (C_4Ferov) (green) and the literature RP phases of Fillafer et al.[1] (yellow). Both PXRD show similar 100-pattern but shifted due to a different interlayer distance.

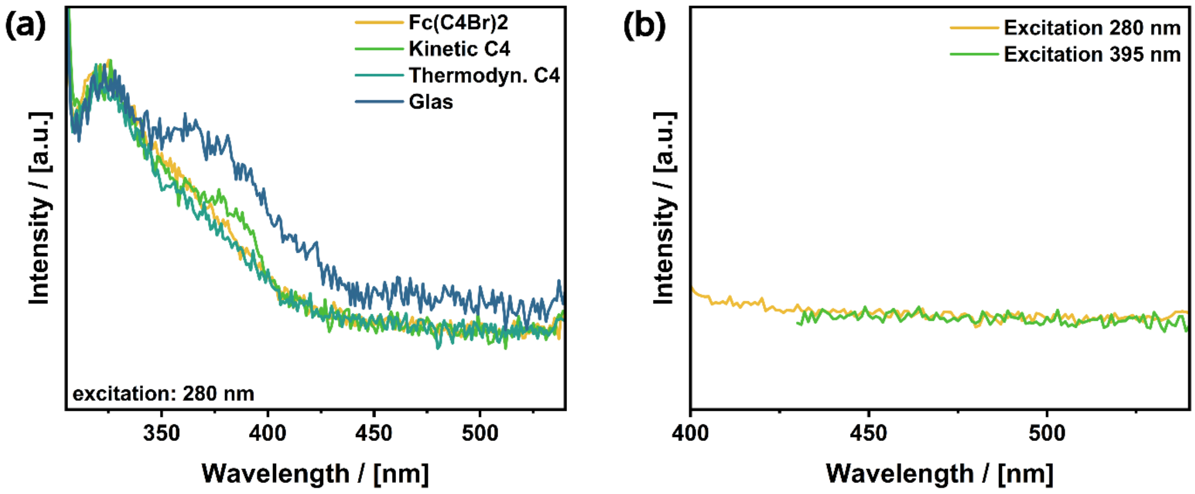

Figure S16. Fluorescence spectra of a) the 'k' (green)-, 'e'-C4Ferovs (turqious) and the C_4-Fc molecule (yellow) as well as the glass slide reference (blue) at 280 nm/4.43 eV, showing no signal beside the glass. b) Excitation of the 'e'-Ferovskite at two different wavelengths (4.43 eV (yellow) and 3.14 eV (green)) with no signal in the relevant range for the ferrocene.

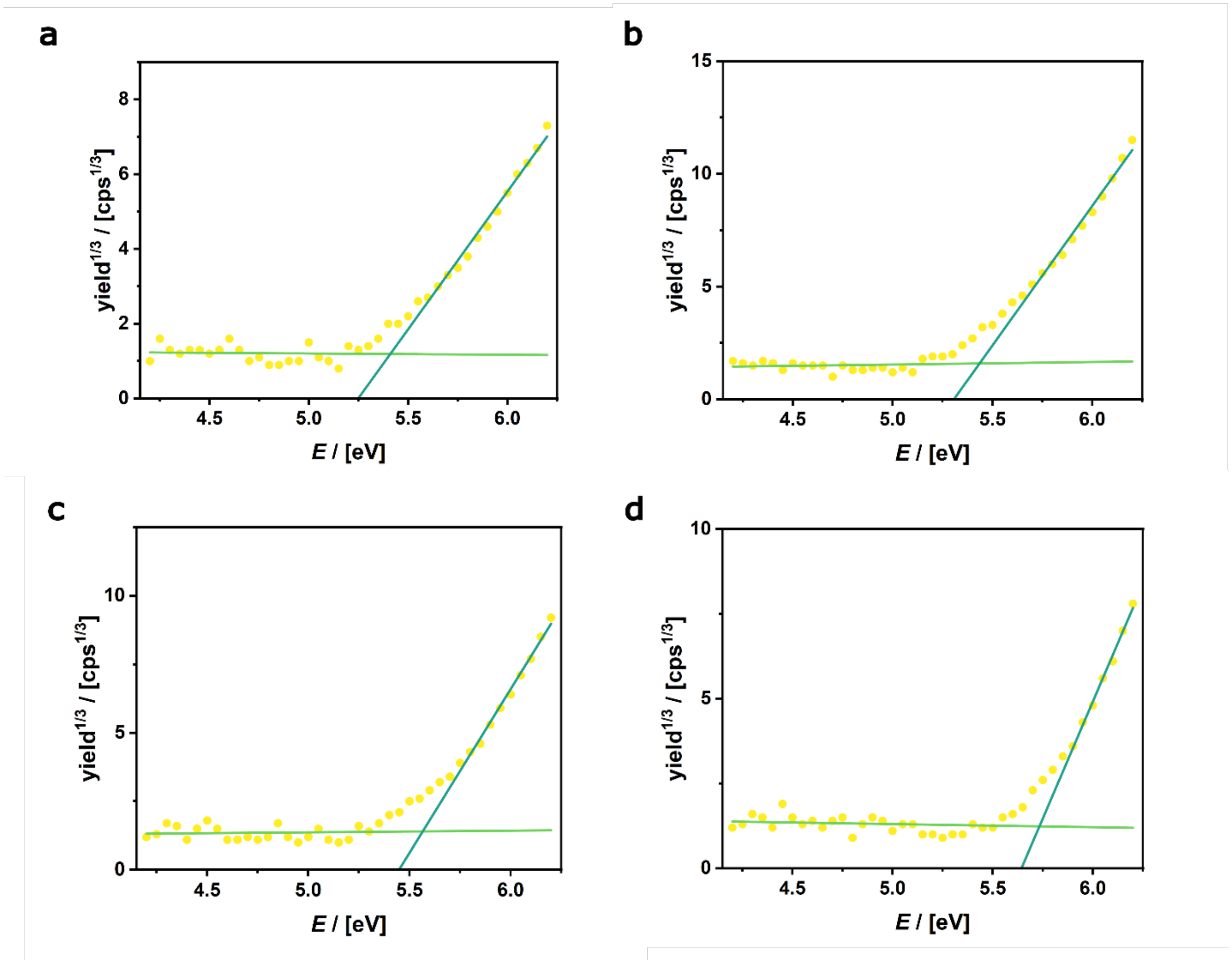

Figure S17. a-d) Photoelectron spectroscopy in air (PESA) of the 'k'-C_3-C_6 ferrovskite phases. The valence band edge values were determined by linear regression of the yield increase for all phases with C_3: -5.50 eV, C_4: -5.49 eV, C_5: -5.60 eV, C_6: -5.74 eV.

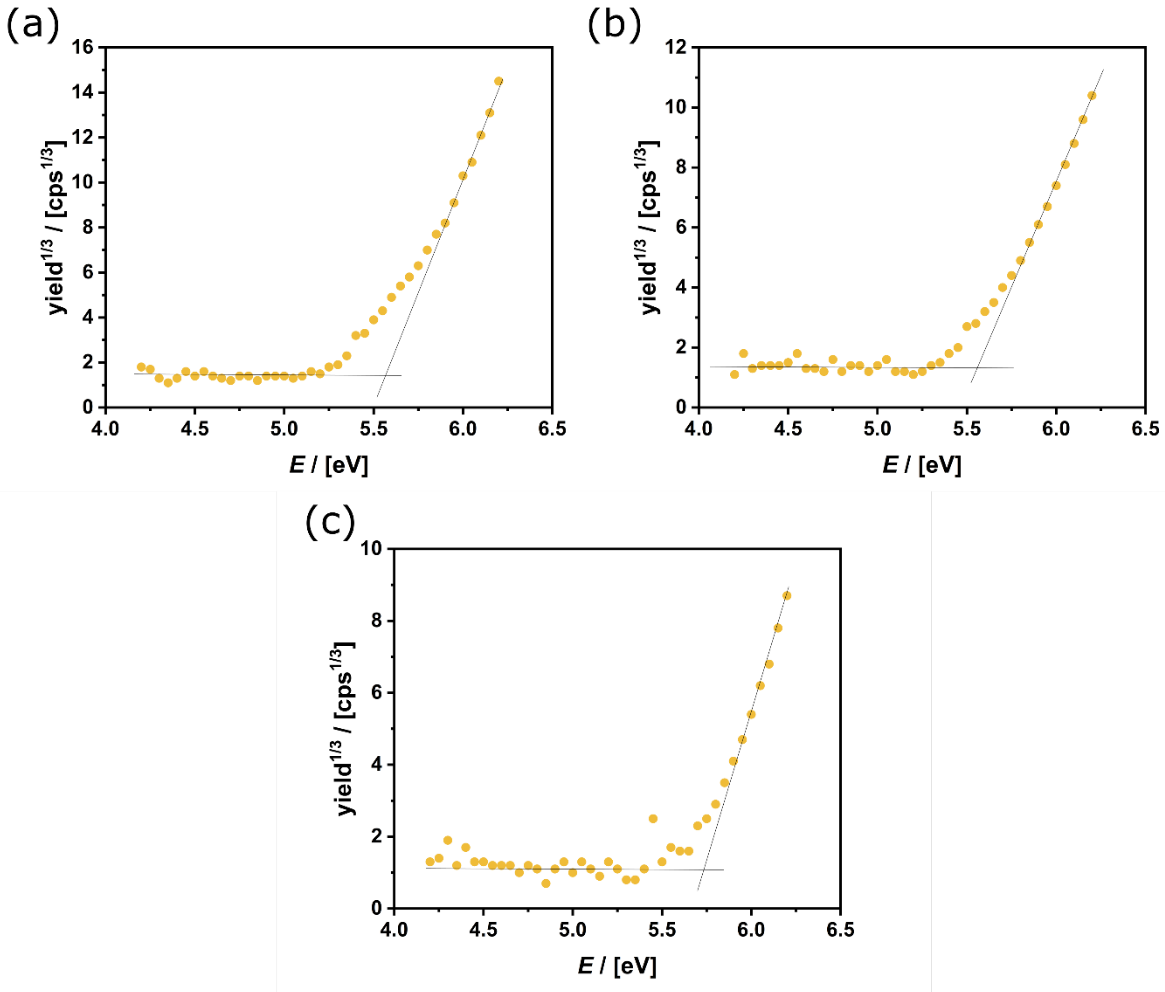

Figure S18. Photoelectron spectroscopy in air (PESA) of the a) 'e'-DJ- b) 's'-C_4 ferrovskite RP phases and c) C_4 Fc-derivative. The 'e' phase shows a valence band edge of -5.59 eV, the 's'-RP phase of -5.58 eV and the free Fc-derivative of -5.70 eV. All values were determined by linear regression of the yield increase.

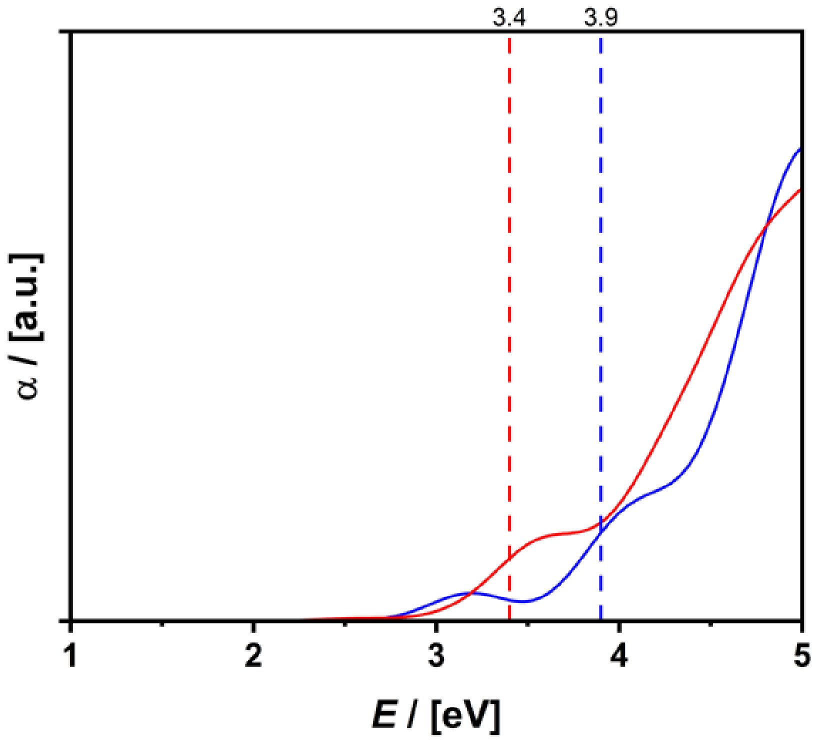

Figure S19. Comparison of the calculated absorption coefficients of the 'e'- (red) and 'k'- (blue) C_4 ferrovskite phase with highlighted band gap of the lead bromide phase. The exact values are red shifted, but the calculated trend is in agreement with the experimental data.

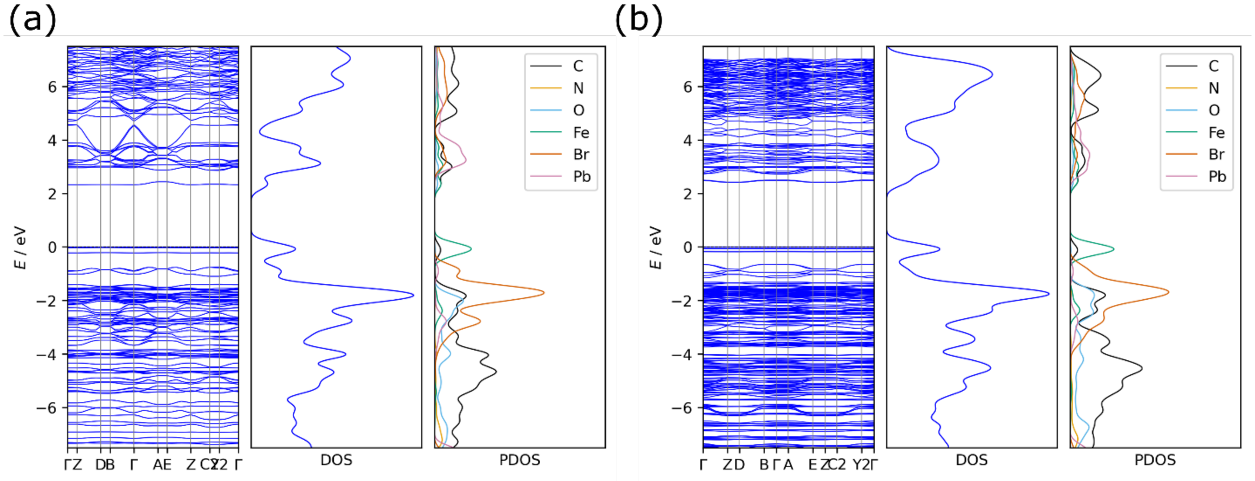

Figure S20. DFT calculations using the generalized gradient approximations (GGA) by employing the PBEsol XC functional show the band structures of the 'k'- (a) and 'e'- (b) C_4 ferrovskite phase with the ferrocene orbitals determining the VB and CB positions.

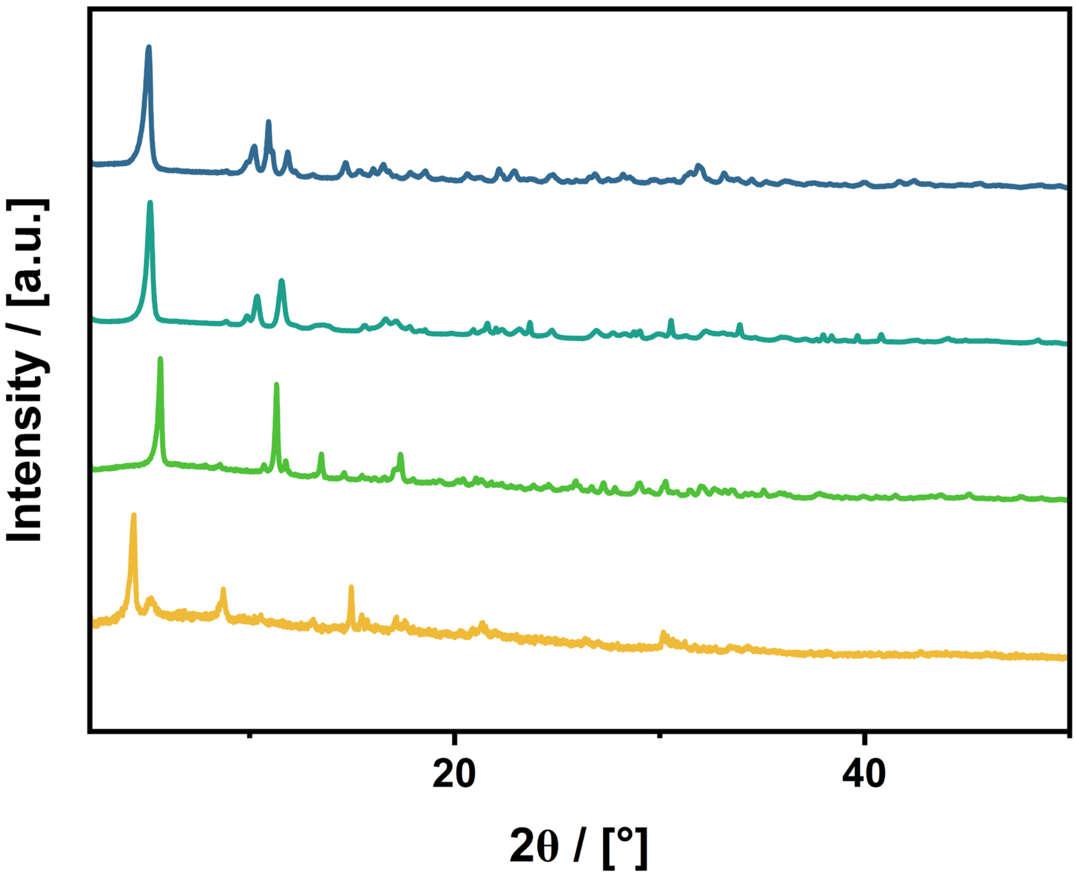

Figure S21. PXRDs of the 'e'-C_nFerovs. C_3: yellow, C_4: green, C_5: turquoise, C_6: blue.

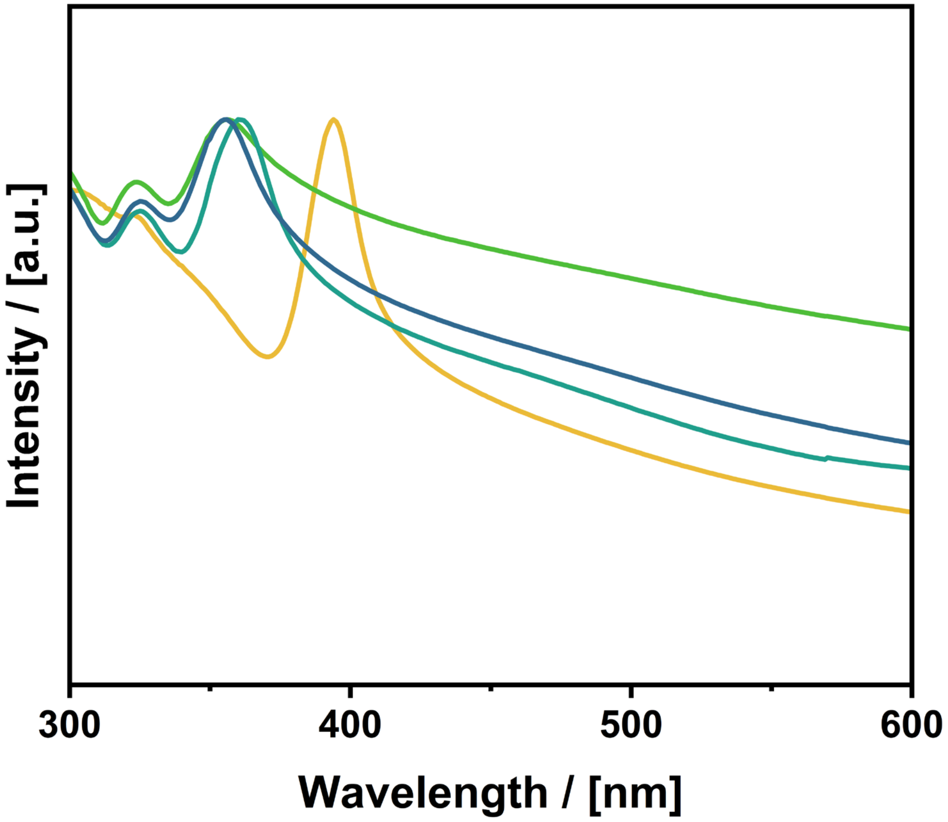

Figure S22. UV-Vis spectra of the 'e'-C_nFerovs with chain lengths C_3-C_6. C_3: yellow, C_4: green, C_5: turquoise, C_6: blue. All ferrovskites exhibit exciton peaks typical for layered materials.

References

(1) Fillafer, N.; Kuper, H.; Schaate, A.; Locmelis, S.; Becker, J. A.; Krysiak, Y.; Polarz, S. Design of Active Defects in Semiconductors: 3D Electron Diffraction Revealed Novel Organometallic Lead Bromide Phases Containing Ferrocene as Redox Switches. *Advanced Functional Materials* **2022**, *32* (24), 2201126. https://doi.org/10.1002/adfm.202201126.
